# Evaluation of the Sensitivity of Proteomics Methods Using the Absolute Copy Number of Proteins in a Single Cell as a Metric

**DOI:** 10.3390/proteomes9030034

**Published:** 2021-07-20

**Authors:** Benjamin C. Orsburn

**Affiliations:** Department of Pharmacology and Molecular Sciences, Johns Hopkins University, Baltimore, MD 21205, USA; borsbur1@jhmi.edu

**Keywords:** proteomics, protein informatics, mass spectrometry, single cell, copy number

## Abstract

Proteomic technology has improved at a staggering pace in recent years, with even practitioners challenged to keep up with new methods and hardware. The most common metric used for method performance is the number of peptides and proteins identified. While this metric may be helpful for proteomics researchers shopping for new hardware, this is often not the most biologically relevant metric. Biologists often utilize proteomics in the search for protein regulators that are of a lower relative copy number in the cell. In this review, I re-evaluate untargeted proteomics data using a simple graphical representation of the absolute copy number of proteins present in a single cancer cell as a metric. By comparing single-shot proteomics data to the coverage of the most in-depth proteomic analysis of that cell line acquired to date, we can obtain a rapid metric of method performance. Using a simple copy number metric allows visualization of how proteomics has developed in both sensitivity and overall dynamic range when using both relatively long and short acquisition times. To enable reanalysis beyond what is presented here, two available web applications have been developed for single- and multi-experiment comparisons with reference protein copy number data for multiple cell lines and organisms.

## 1. Introduction

Researchers who are interested in analyzing the global expression of protein have more options than ever before, due to a flurry of developments in proteomics technologies over the last 20 years [1]. Today, most proteomics work is performed using liquid chromatography-coupled tandem mass spectrometry (LCMS). While a few groups use LCMS successfully to analyze intact proteins via top-down proteomics, most work today is LCMS of proteolytically digested proteins, which is often referred to as shotgun proteomics [2]. With a dizzying number of hardware platforms, reagents, and methodologies to choose from, it is natural that researchers promote their favorite technology. Metrics for the performance of different methods do exist, with relative numbers of peptide and protein identifications per unit time being a metric of choice. A challenge in evaluating peptide and protein counts, as an objective metric for overall method performance, is in the number of variables that can be altered in the data processing pipelines that can affect these results. For example, utilizing a larger potential database to compare shotgun proteomics data to invariably increases the number of peptide identifications [3]. Increasing the search space further, to evaluate an increasing number of biologically likely post-translational modifications, will have a similar effect [4,5,6].

One metric of note is the “proteomics ruler”, developed by Wizniewski et al., and enabled as an add-in feature in the Andromeda post-search analysis bioinformatics package [7,8]. The proteomics ruler uses the relative quantification data of proteins in a sample and normalizes these abundances to that of the major histones that are present in mammalian cells. Histone proteins exist in a tightly conserved ratio in mammalian systems and directly proportional to the DNA present in a cell. Furthermore, the amount of DNA in a cell is an extremely consistent value. The proteomics ruler leverages these values as constants and can generate a remarkably accurate estimate of the absolute concentration of each protein in the cells analyzed. By comparing this concentration to the protein size, it is possible to estimate the number of copies of each individual protein within a single cell of the samples being analyzed. The proteomic ruler has been successfully applied to assess a wide array of samples, from human cancer cell lines through to mammalian organs, and is a valuable tool in shotgun proteomics today.

While the proteomics ruler has been employed in multiple studies, by the teams involved in the development of the method, it has not been more widely employed to date. This is likely due, at least in part, to the fact that it is currently only available through the Perseus program. In this review, I will more widely apply the proteomics ruler as a comparison between different proteomic technologies. Despite the ethical concerns regarding the use of HeLa cells in -omics studies, it continues to be actively used by proteomics labs worldwide, and is the most obvious cell line from which to perform this re-analysis exercise [9]. It is worth noting, however, that a recently preprinted interlaboratory study has described a large degree of variation in HeLa cell lines. Gene and protein expression profiles, as well as protein copy numbers, were shown to differ between the 14 HeLa stock samples that were obtained from 13 labs around the world [10]. The results herein should be treated with extreme caution.

To date, the most comprehensive shotgun proteomic analysis of HeLa cells was performed by Bekker-Jennsen et al. [11]. In that study, the proteomic ruler was used, and the protein copy numbers were obtained for over 12,000 distinct protein groups in the cell line. Through use of a simple R scripts and complementary web-based Shiny Apps, developed for this in this work, I will use this heavily fractionated proteome as the base metric. By applying the protein copy numbers derived in this study to the proteins identified by other proteomics techniques, we can obtain a simple visualization of the relative biological sensitivity of that method compared to others.

## 2. Materials and Methods

### 2.1. Obtaining Data

The copy number data for the HeLa cell lines were obtained from the processed output of the original studies obtained from ProteomeXchange partners. A table of the files presented here with references, if publicly available, is shown in Table 1. The identifiers from the processed data from the original studies were used when possible. When processed data were not directly available for comparison, the original vendor files were processed in-house. In-house searching was performed against the UniProt SwissProt database using the appropriate alkylation modification and the oxidation of methionine as the only variable modification. All data dependent files were searched with Proteome Discoverer 2.4 using the MSAmanda 2.0 search engine [12] and Percolator for false discovery rate (FDR) estimation. All default parameters in Proteome Discoverer using the vendor provided workflow templates “PWF_QE_Basic_Percolator” and “CWS_Basic” were utilized unless otherwise noted here. Orbitrap data were searched with a 10 ppm MS1 tolerance and a 0.02 Da MS/MS tolerance if high resolution and 0.6 Da MS/MS if ion trap. Data from TOF instruments were converted to MGF with ProteoWizard and searched using a 50 ppm MS1 and 0.1Da MS/MS tolerance. All data-independent acquisition (DIA) data presented herein are based on the results from the original studies. The protein lists used for the analysis of SOMASCAN data were obtained from published studies [13]. When necessary for previously published data the UniProt identifiers were extracted by pulling the list into R using the TidyVerse and Tabulizer packages [14,15].

### 2.2. Compiling the Absolute Copy Numbers

The UniProt identifier for the best protein identifier was removed from the final protein report from the reference data deposited. The majority protein and protein group identifiers were used from MaxQuant and Proteome Discoverer, respectively. MaxQuant assigned the majority protein to the accession that possesses greater than 50% of the peptides from all proteins with equivalent evidence [16]. Proteome Discoverer utilizes different logic depending on the version in use. In versions 1.0–2.0, the protein group is assigned to the protein with the highest total percentage coverage. In versions 2.1 onward, the largest protein in a group bearing equivalent evidence to all other proteins is assigned [17]. A recent analysis of 22 search engines demonstrated that resulting protein lists change little from the same MS/MS evidence [18], and therefore the two will be considered as equivalent in this light reanalysis.

The processed data from the Bekker-Jennsen et al. study identified 14,238 distinct proteins, of which 14.178 were assigned a copy number estimate based on IBAQ and the Proteomics Ruler Perseus accessory program [11]. I will refer to this as the HeLaHF dataset for the remainder of this work. Table 1 is a summary of the files used in this review.

### 2.3. Visualization of Copy Number Distribution in R/Shiny

The copy numbers of the Proteome Ruler and those applied to the identifiers are plotted using the base R histogram functions utilizing 30 bins and plotted versus number of total protein counts as well as normalized to density [27]. All work was performed in R studio. The R script is publicly available at https://github.com/orsburn/copynumbeR (accessed on 14 July 2021). The Shiny Apps files presented in this work and additional Proteomic Ruler base datasets can be found at https://www.lcmsmethods.org/methodtesting (accessed on 15 April 2021). All files utilized in this review were obtained from ProteomeXchange partner repositories as referenced in the Supplemental Information [28].

## 3. Results

### 3.1. Generational Improvements in Proteomics Hardware for Data Dependent Analysis

**Figure 1:**
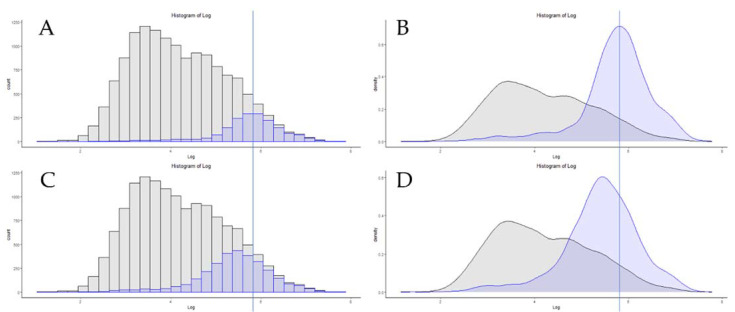
The copy number distribution of two identical sample injections and chromatography systems on a Q-Exactive classic by protein counts (**A**) and density (**B**), compared to a Q-Exactive HF system (**C**,**D**). The vertical line represents the apex copy number on the classic system.

One of the most powerful forces driving the growth of proteomics as a field has been the increase in LCMS hardware performance over time. For a more thorough review of this topic, please see “The One Hour Yeast Proteome”, which thoroughly covers this topic up to the date of its publication [29]. A clear example of this increase in performance is the step from the original Q-Exactive system to the Q-Exactive high-field (HF) system. Several improvements in the architecture of the HF system exist over the “Classic” system, and these have been thoroughly described by others [20,30]. A more refined series of lenses, segmented quadrupoles with more symmetrical isolation efficiency and a lower instrument overhead, undoubtedly have effects on instrument sensitivity. However, these changes were implemented in the Q-Exactive “Plus” system as well. In this author’s hands, the “Classic” and “Plus” have a similar performance for global proteomics (data not shown). However, replacing the larger D30 Orbitrap system for the smaller diameter D20 Orbitrap has a marked change in the overall instrument performance. By increasing the curvature of the electric field in the D20 system, spectra of the same resolution can be obtained in approximately half the time of the earlier design, effectively doubling the spectral acquisition rate [31]. Typical results from these two systems, utilizing a 15 cm PepMap column and identical HPLC systems for 200 ng of HeLa digest standard, are 2000 proteins for 120 min for the classic and 3400 proteins for the HF (Table 1). Figure 1A,C represent the distribution of the absolute copy numbers of proteins found in HeLa cells in grey, with the distribution of the proteins from the classic and HF devices, respectively, in blue. A vertical line was added for reference, to flag the apex of the copy number counts for the “Classic” system. The 1400 additional proteins that were identified by the HF, using this identical sample and chromatography system, predominantly belong to lower copy number protein groups. Figure 1B,D represent the normalized density distribution of these same numbers, with the vertical bar again indicating the apex reference point of the “Classic” system.

### 3.2. Use of Absolute Copy Number for Optimization of Chromatographic Conditions

**Figure 2:**
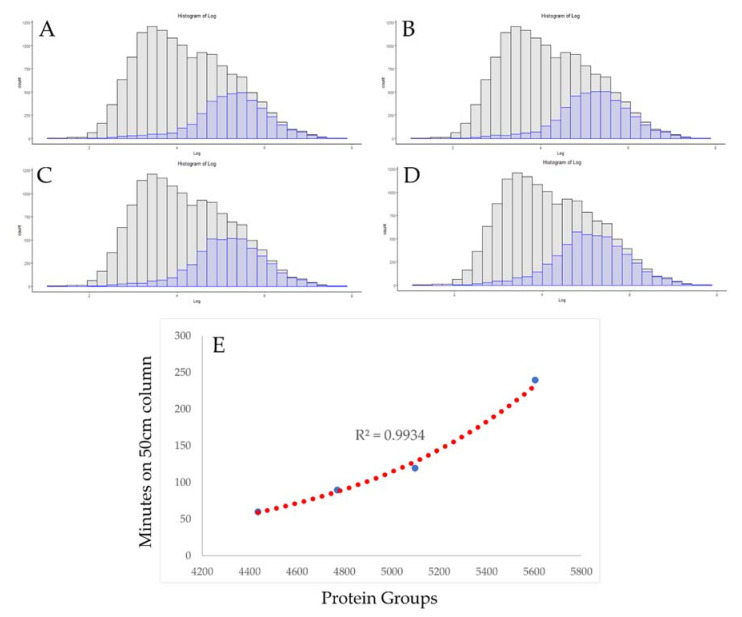
Visualizing diminishing returns in gradient extension. The distribution of protein copy numbers on an Orbitrap Fusion Lumos with the same chromatography conditions and utilizing the instrument’s highest scan acquisition rates with a total gradient length of 60, 90, 120, and 240 min (**A**–**D**, respectively). (**E**) A plot of the number of proteins identified versus total gradient time demonstrating the exponential increase in run time required to improve coverage under these conditions.

A comprehensive optimization study of the Orbitrap Fusion II “Lumos” instruments, by Espadas et al., included work with gradient optimization on 50 cm columns using 1000 ng injections of HeLa digest. Table 1 includes a summary of the protein identifications that were obtained in the work, using 60, 90, 120, and 240 min gradients. At 60 min, the instrument identified an impressive 4475 protein groups and 240 min increased this number to 5604, with other times falling in-between these two points. As shown in Figure 2, increasing the length of the gradient did, in every case, increase the number of proteins that were identified, as well as leading to the discovery of lower copy number proteins. The increase in protein identifications is clearer when taking the protein numbers into account, rather than by visualizing in this manner, because the distribution of 10% of the proteome across 30 bins appears slight to the eye, demonstrating a limitation of copy number visualization. A better metric can be obtained by considering the increase in identified proteins per unit time, as shown in Figure 2E. By increasing the gradient time, more proteins are identified, but with decreasing returns per unit time. While 300 protein groups may be added by extending the run time from 60 to 90 min, to identify an additional 600 protein groups to the number acquired with a 120 min gradient, the total run time must double. While there are limited studies in the literature to observe in this manner, this does appear to be a trend that is instrument- and chromatography-specific. Table 1 includes the summary of three files from an Orbitrap Elite system generated by the authors. In our hands, the Orbitrap Elite appears to have a maximum coverage of approximately 4000 protein groups, almost regardless of the gradient length and scan type utilized. The only single-shot runs that have exceeded 4200 protein groups utilized a 75 cm column and a 540 min gradient, more than 2× the amount of time necessary to identify 3900 unique protein groups from 200 ng of HeLa digest sample.

### 3.3. Rapid Proteomics Methods

**Figure 3:**
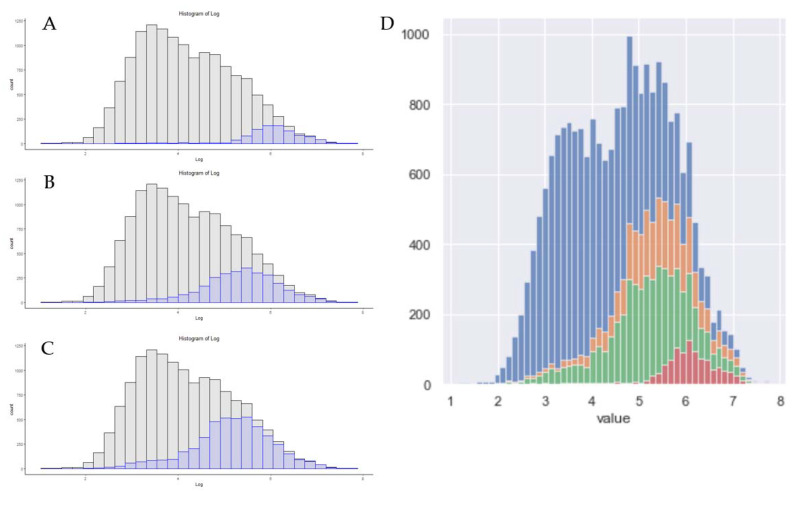
The distribution of protein absolute copy numbers in three methods of less than 30 min in total length. (**A**) An Orbitrap Velos 30 min gradient. (**B**) Results from a 21 min analysis on a Q-Exactive high-field system. (**C**) A 21 min gradient utilizing a FAIMS-equipped Exploris 480 system with a single compensating voltage of −70 EV. (**D**) An overlay of these data using a 60 bins with (**A**) in red, (**B**) in green and (**C**) in orange.

A common and accurate criticism of proteomics technology is the speed at which data could be obtained [32,33]. With improvements in mass spectrometry, chromatography material, and sample preparation techniques, progress has been made toward shortening the total time between the samples being obtained and analyzed. In both the commercial sector and in clinical sciences, both high-flow proteomics and capillary separation are utilized to increase both the up-time and speed of analysis [34,35,36,37]. Nanoflow is still the most utilized separation method in proteomics, but efforts are underway to decrease nanoLC run times [38]. As a summary of recent developments, Figure 3 is a visualization of three generations of Orbitrap instruments, and the results obtained when analyzing samples with gradients less than 30 min in length. The Orbitrap Velos file used nanoflow liquid chromatography and parallelized use of the Orbitrap for MS1 and ion trap for MS/MS, and achieved identification of 1171 protein groups in this reanalysis. Recent work using shorter gradients on high-field Orbitrap systems, demonstrates the progress in hardware performance over the last 10 years. The use of a chromatography system with rapid pre-formed gradients, the EvoSep1, demonstrated remarkable coverage when used for acquisition on a Q-Exactive HF system (Figure 3C) [38]. Building on the EvoSep technology, a high-field asymmetric waveform ion mobility spectrometer (FAIMS), equipped Exploris 480 system, identified 3182 proteins with high-resolution MS/MS spectra and a single FAIMS compensating voltage of −70 EV (Figure 3C) [23]. While alterations at this level are reasonably easy to visualize, it is important to consider how scaling and binning can affect any visualization. As an example, Figure 3C demonstrates an overlay of these same results when using twice the number of bins.

### 3.4. Absolute Sensitivity in Single-Shot Proteomics Today

**Figure 4:**
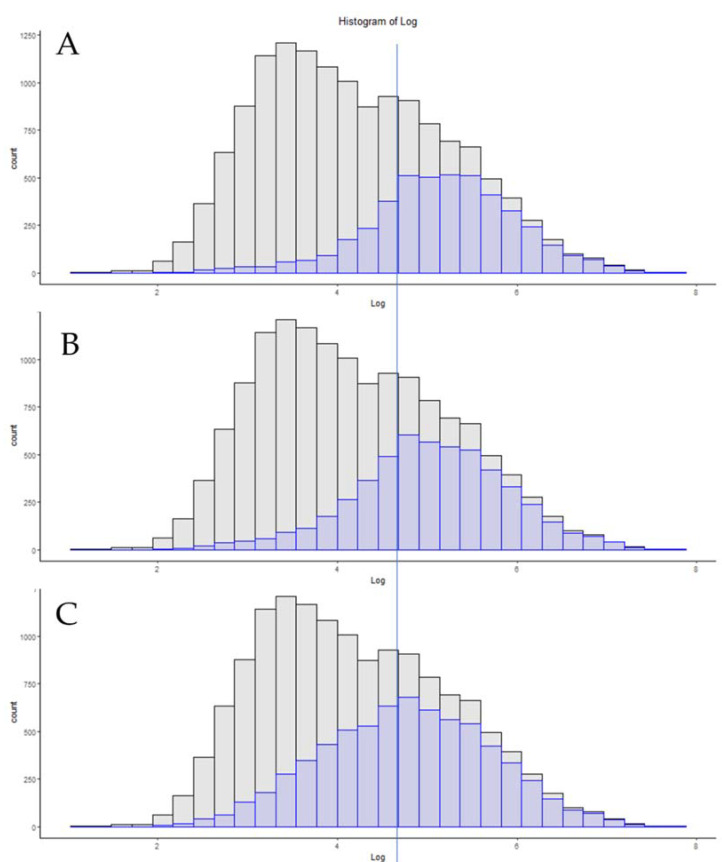
A comparison of three of high-coverage proteomics methods utilizing a 2 h total run time. The vertical line is used here as a reference point to help visualize the shift in copy number distribution toward the apex of the deepest coverage method. (**A**) Lumos system with 1000 ng injection and 50 cm column. (**B**) TIMSTOF Pro system operating in pasefDDA mode. (**C**) A modified TIMSTOF operating in pasefDIA mode.

The field of proteomics has been almost impossible for insiders to keep up with, as new methods appear even more frequently than new hardware [39]. Recently, new hardware designs that are leveraging sophisticated ion mobility devices have appeared, which have challenged the status quo of Orbitrap dominance in proteomics. Figure 4 is a comparison of the highest coverage single-shot analyses that the authors have seen to date. The files shown are from published studies using 2 h gradients, where A is a 1000 ng injection of HeLa digest ion an Orbitrap Fusion 2 “Lumos” system. Figure 4B is a file from a TIMSTOF Pro system, and Figure 4C is from the recently published results on the TIMSTOF system operated in pasefDIA. The Lumos achieves an impressive 5098 protein groups when reprocessed with MSAmanda 2.0 for this comparison. The TIMSTOF Pro system, when analyzed in the same manner, achieves 5970 protein groups using the same software [22]. The recently published data using pasefDIA achieves a remarkable 7699 protein groups when processed by the authors in the original study, which is a number that is over 54% of the total proteome reference numbers [40].

### 3.5. Match between Runs

**Figure 5:**
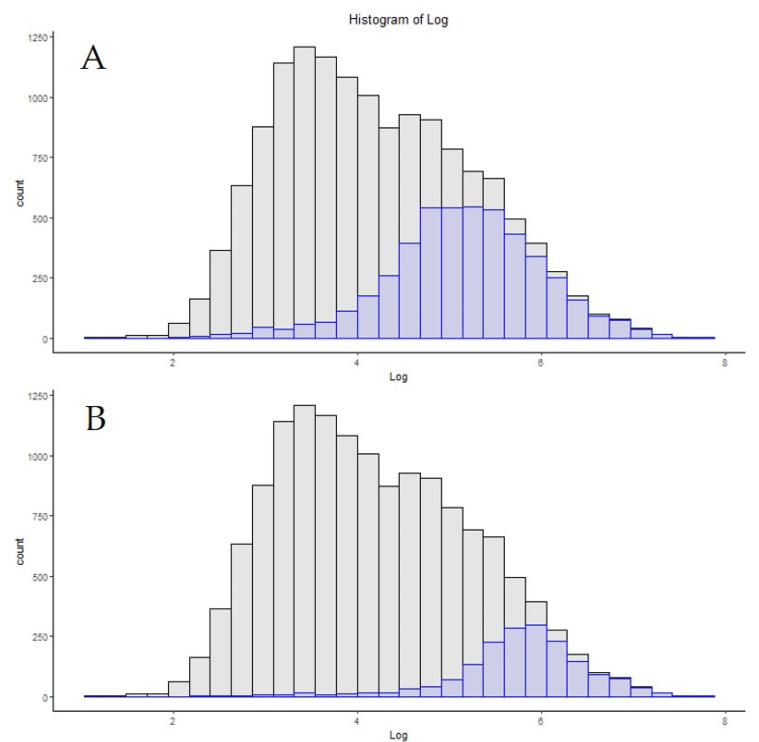
A comparison between (**A**) protein groups identified in a 60 min BoxCar run when match between runs is used on a comprehensive MS1 library. (**B**) The same file when match between runs is not employed.

Match between runs (MBR) allows proteomics results to be additive in nature, with identifications made by MS/MS in one run to be applied to a second run if the chromatographic features and isotopic profile match within set parameters [41]. A recent method, called BoxCar, leans heavily on MBR. In BoxCar, multiple MS1 scans are gas-phase fractionated to obtain a more democratic distribution of MS1 signal. By collecting multiple fractions and capping each gas-phase fraction at a set limit, it is more difficult for high-abundance ions to fully fill the Orbitrap, and therefore suppress the signal of other coeluting ions. BoxCar results in an increase in the signal-to-noise ratio of lower abundance ions, at a cost of cycle time, due to the amount of time spent acquiring MS1 scans [25]. Figure 5 is an example of the results obtained from a 60 min BoxCar injection of 500 ng of HeLa lysate. When MBR is employed, BoxCar on Q-Exactive HF system can identify 6479 protein groups when matched against a highly fractionated DDA library that is generated on the same system. Figure 5B shows the number of those proteins that are identified in the absence of an MS1 library or MBR. Methods that are derived from BoxCar that utilizes parallelization in the ion trap on a Tribrid device and BoxCarDIA have demonstrated promise in alleviating the relative cycle time hits from the original method [42,43]. In addition, recent work has demonstrated a mechanism for estimating false discovery rates in MBR, which could go a long way toward realizing the potential of this strategy [44,45].

### 3.6. Single-Cell Proteomics

**Figure 6:**
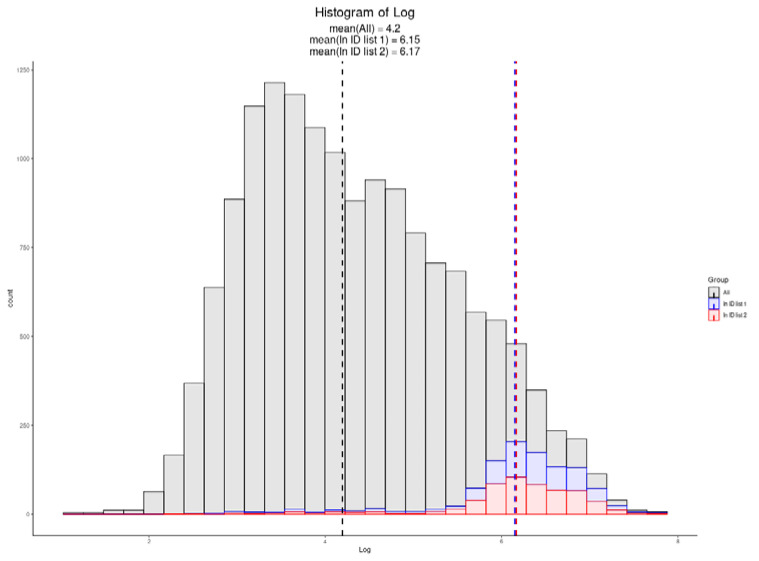
The copy number distribution of protein copy numbers identified in two single-cell methods. A TMT experiment consisting of single cells with a carrier channel of 20 cells is shown in blue. A label-free analysis of a single HeLa cell is overlaid in red. Apexes are defined by color and the mean log copy for each single-cell run is provided.

During the review of this text, a preprint from Hartlmayr and Ctortecka et al. demonstrated single-cell proteomics, utilizing a novel semi-automated platform coupled to a FAIMS-equipped Exploris 480 system [26]. The authors used this platform to analyze single cells, including those prepared from a HeLa cell line. This timely study allows a more direct analysis of the relative accuracy of the proteomic ruler data itself, as well as captures an understanding of the current state of single-cell proteomics technology today. Both label-free proteomics and TMT-labeled proteomics, utilizing carrier channels in a similar manner to SCoPE, were performed. As shown in Figure 6, both the methods obtained similar coverage, with a single unlabeled cell resulting in 608 protein groups, while single cells that were loaded with a 20 cell carrier channels resulted in 769 protein groups in our reanalysis. While altogether remarkable that such a depth of coverage can be obtained from single human cells, the fact that the distribution does not appear fully biased to the single highest calculated copy number proteins hints at some level of uncertainty in the proteomic ruler data. As single cells are prepped in a much different manner than bulk cell homogenates, and with the recently described variability in HeLa cell cultures globally, this may be altogether unsurprising.

### 3.7. Additional Methods

SomaScan is a commercially available alternative to LCMS proteomics workflows which utilized nucleic acid aptamers. 

**Figure 7:**
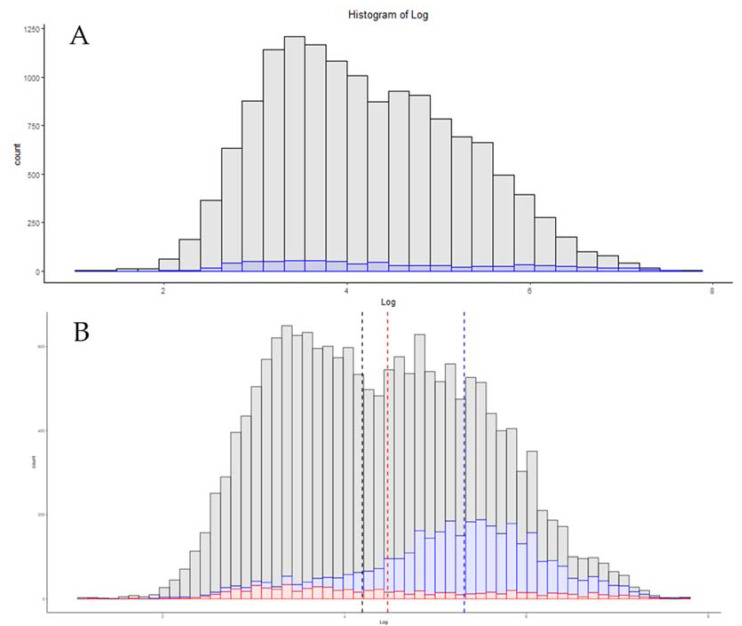
(**A**) The distribution of SomaScan 1300 targets by copy number by count. (**B**) Comparison of this distribution against a recently published 21 min method utilizing an Exploris 480 equipped with FAIMS with 60 bins utilized to increase the granularity of visualization.

SomaScan aptamers are arranged in multiple configurations, with the SomaScan 1300 containing the highest relative number of targets. Figure 7 is a comparison of the 1300 kit and targets against the HeLa HF copy number library [19]. Despite the relatively small number of targets compared to any modern LCMS-based proteomics method described in this work, this alternative technology targets proteins across the entire dynamic range. The median log copy number of proteins in the SOMASCAN 1300 kit is 4.47, giving it one of the deepest dynamic range distributions of any technology reviewed here. However, as shown in Figure 7B, the small number of relative targets is dwarfed by even a 21 min LCMS method, utilizing some of today’s best hardware, such as a FAIMS-equipped Exploris 480.

The Supplemental Material contains copy number distribution data from all the files described in Table S1.

## 4. Conclusions

Today, we still do not have a complete picture of what the proteome is, and it seems likely that shotgun proteomics may never be able to fully answer critical questions, such as “how many human proteoforms are there?” [46]. With proteoforms being the next currency in proteomics, top-down technology will need to continue to develop at a rocket pace to eventually pick up the slack [47]. Shotgun proteomics will not be going away anytime soon, and the tools have continued to mature to a point where we are competitive in coverage, time, and sensitivity with today’s genomics and transcriptomics techniques [48]. Proteomics, as a field, still has plenty to overcome, most notably standardizing sample preparation and methodology, and the maturation of informatics. When we control for sample preparation, LCMS-based proteomics has demonstrated remarkable intra- and inter-lab reproducibility [49]. As a growing and maturing field, with diverse biological problems to confront, method development and optimization will need to continue until all organisms and organelles have been successfully tackled. The goal of this review was to take a snapshot of where we are today, and to use visualization of protein copy number depth as an additional tool when making inevitable decisions.

New hardware is released by instrument vendors nearly every year. If the goal of a lab is to obtain deeper proteomic depth with no further alteration in workflows, moving up to the newer generation of hardware may be the best solution for that task. On the same hardware, increasing the LCMS gradient time should almost always lead to increases in the peptide and protein numbers, but, as shown in Figure 2, this may be a slope of steeply diminishing returns. For some projects, increasing the total acquisition time from 60 min to 240 min may be an acceptable solution. For commercial labs or those with more users than hardware, that may be too steep of a price for an additional 1000 protein identifications. A common criticism of proteomics has always been the relatively low number of samples in each study, relative to genomics or transcriptomic studies [50]. As such, some groups are under pressure to use shorter acquisition times to address clinical cohorts, and several of today’s hardware advances appear well-suited to these tasks (Figure 3). Today’s current-generation instruments can identify over 3000 proteins from a HeLa standard in as little as 21 min of total run time, nearly three times the number of slower instruments of previous generations (Figure 4).

We must also consider the quality of the evidence that we consider acceptable for making a peptide or protein identification. While some groups have argued in the past, [33] and some more recently [51], that a high-resolution mass and retention time is sufficient to assign a peptide identification, it is fair to say that this is not universally accepted today. However, peptide identification that is supplemented with algorithms such as match between runs (MBR) is becoming increasingly adopted, and may be essential in some cases to meet the increasing expectations of collaborators. With deep analyses of the accuracy of these tools [44], providing reasons for optimism, and the recent description of a method for false discovery rate estimation for MBR, MS1-based peptide identification may soon experience a renaissance [45]. The recently described BoxCar method for quadrupole Orbitrap systems leans more heavily on MBR than any previously described technique. By sacrificing the number of MS/MS scans per run, to obtain more MS1 scans with improved signal-to-noise ratios, peptides identified by the former decrease markedly (Figure 5). Without the use of tools that can perform MBR, or without MS1 libraries, BoxCar can appear to be a waste of time and effort. The increase in the signal-to-noise ratio is too tempting of a target, however, and work continues to develop methods based on these innovative methods [42,43]. BoxCar is not the only method today that sacrifices the number of MS/MS scans per LCMS run to obtain alternative data. The FAIMS front end for current-generation Orbitrap instruments can be operated at multiple compensation voltages (CV) in each run. Although each CV requires an MS1 scan and a corresponding decrease in the available MS/MS acquisition time, this gas-phase fractionation leads to overall increases in protein identifications [23].

One of the fastest growing areas in proteomics today is the application to single cells [52,53]. The LCMS community appears to be divided into two distinct camps, those leveraging the newest hardware advances to increase sequencing depth in unlabeled samples [54] and those utilizing chemical tags to amplify peptide signals [55,56,57]. Today, both techniques appear to be able to identify a few hundred proteins per cell, with each innovation adding just a bit more to the overall depth [58]. With a better understanding of today’s limits, such as the maximum loading channels that should be used in amplification-based experiments, further advances will continue to chip away at these limits [59]. One key limitation is the sample preparation, and two preprints that were posted in early 2021 have demonstrated nanoliter semi-automated workflows to address these limitations [26,60]. These are reaping obvious dividends, resulting in some of the most comprehensive single-cell proteomes described to date (Figure 6). With a technology of such focus that can realistically only obtain data on the highest copy number proteins, every advance should move these histograms toward distributions further to the left.

Finally, although LCMS has had a monopoly on proteomics for decades, this is clearly no longer the case. New technologies are appearing today to challenge the status quo in more directly measuring organism phenotypes. SOMASCAN is one early example that has continued to gain ground. The use of aptamer technology appears to be less biased by the absolute protein copy numbers in a cell than LCMS technology (Figure 7). As these arrays continue to increase in the number of probes that may be utilized per sample, SOMASCAN may provide a complementary technology for the identification of proteins that are the most difficult for LCMS.

Today, we can choose from a variety of tools for proteomics, each with its own strengths and weaknesses. Our hope is that this review provides some insight into where proteomics is today. Furthermore, I hope that the tools created in the construction of this manuscript can be helpful to other researchers as they make inevitable decisions and compromises.

I hope that this review helps provide some biological perspective of the sensitivity of proteomics in use today. Further methods are compared in the Supplemental Information. To help facilitate further investigation in this regard, the simple tools used in this work have been made publicly available at www.lcmsmethods.org/methodtesting (accessed on 14 July 2021).

## Figures and Tables

**Table 1:** A summary of select files described in this text with references to the original study. A more complete table of studies and files analyzed is available as supplemental information.

File Description	Number of Proteins	Mean Log Copy Number
HeLa HF 2018 (23 h) [11]	14,179	4.2
SomaScan 1300 [19]	1308	4.47
QE Classic 200 ng 120 min [20]	2016	5.72
QE HF 200 ng 120 min [20]	3487	5.4
Lumos HCD-IT 60 min [21]	4435	5.25
Lumos HCD-IT 90 min	4770	5.21
Lumos HCD-IT 120 min	5098	5.17
Lumos HCD-IT 240 min	5604	5.09
Velos OT-IT 30 min (PRIDE PXD011070)	1171	5.98
TIMSTOF Pro pasefDDA 120 min [22]	5970	5.04
Exploris 480 FAIMS 21 min [23]	3182	5.32
pasefDIA 120 min [24]	7699	4.77
QE HF BoxCar 1 ug 60 min (MBR) [25]	6479	5.16
QE HF BoxCar 1 ug 60 min (MS/MS) [25]	2505	5.77
Exploris 480 Single-Cell TMT 20× Carrier [26]	769	6.15
Exploris 480 Single-Cell LFQ [26]	608	6.17

## Supplementary Materials

The following are available online at https://www.mdpi.com/article/10.3390/proteomes9030034/s1, Supplemental Table S1: the peptide and protein counts of other files analyzed in this review. Supplemental Figures: plotted copy numbers of other files in this review.
