# Supplementary material for: Evaluation of the Sensitivity of Proteomics Methods Using the Absolute Copy Number of Proteins in a Single Cell as a Metric
Source: Proteomes. 2021 Jul 20;9(3):34. doi: 10.3390/proteomes9030034 (PMC8293326; doi:10.3390/proteomes9030034)
Supplement: Supplementary file 1 [file proteomes-09-00034-s001.zip › SupplementalFigures.pptx]

## Slide 1
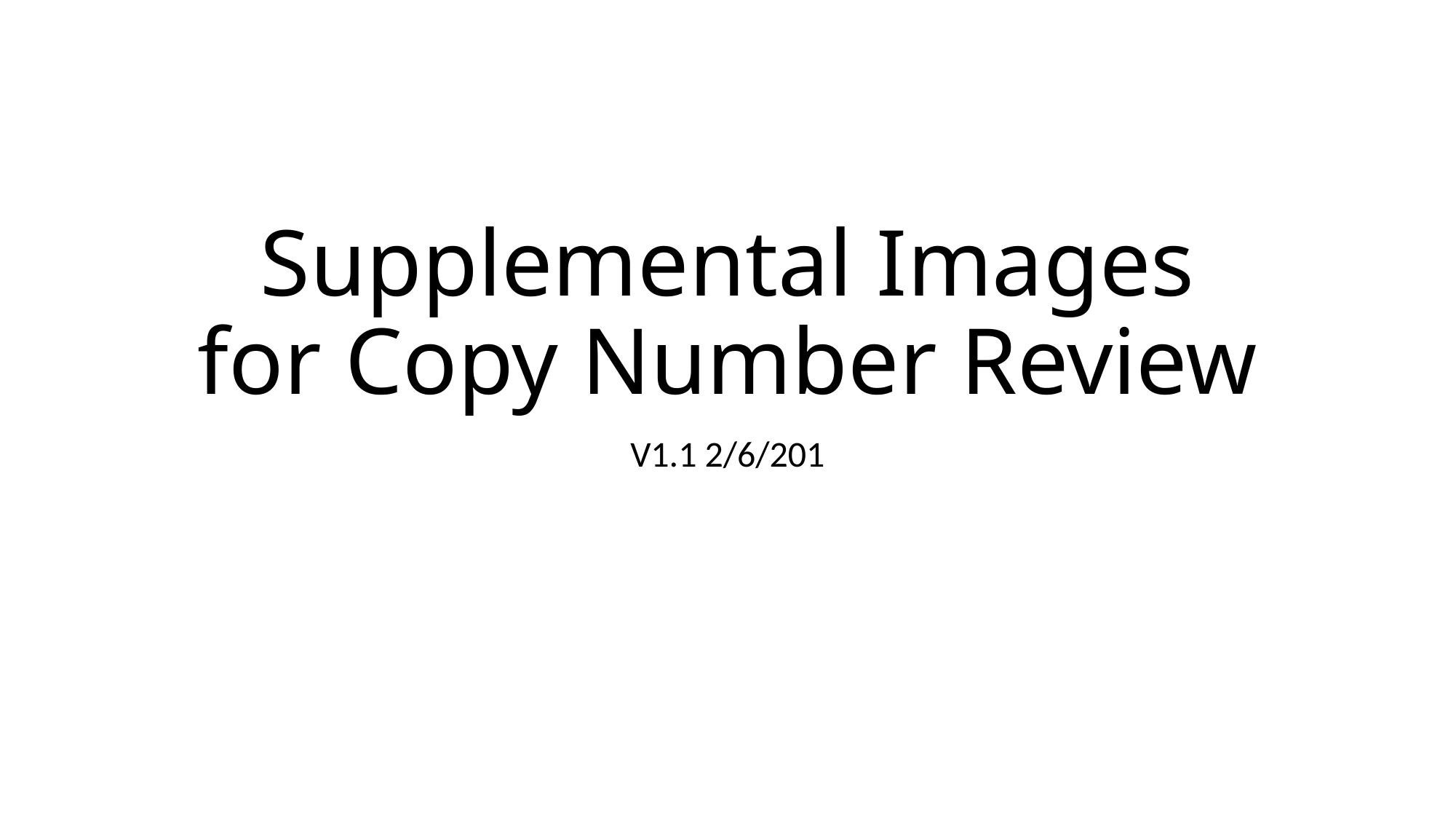

# Supplemental Images for Copy Number Review
V1.1 2/6/201

## Slide 2
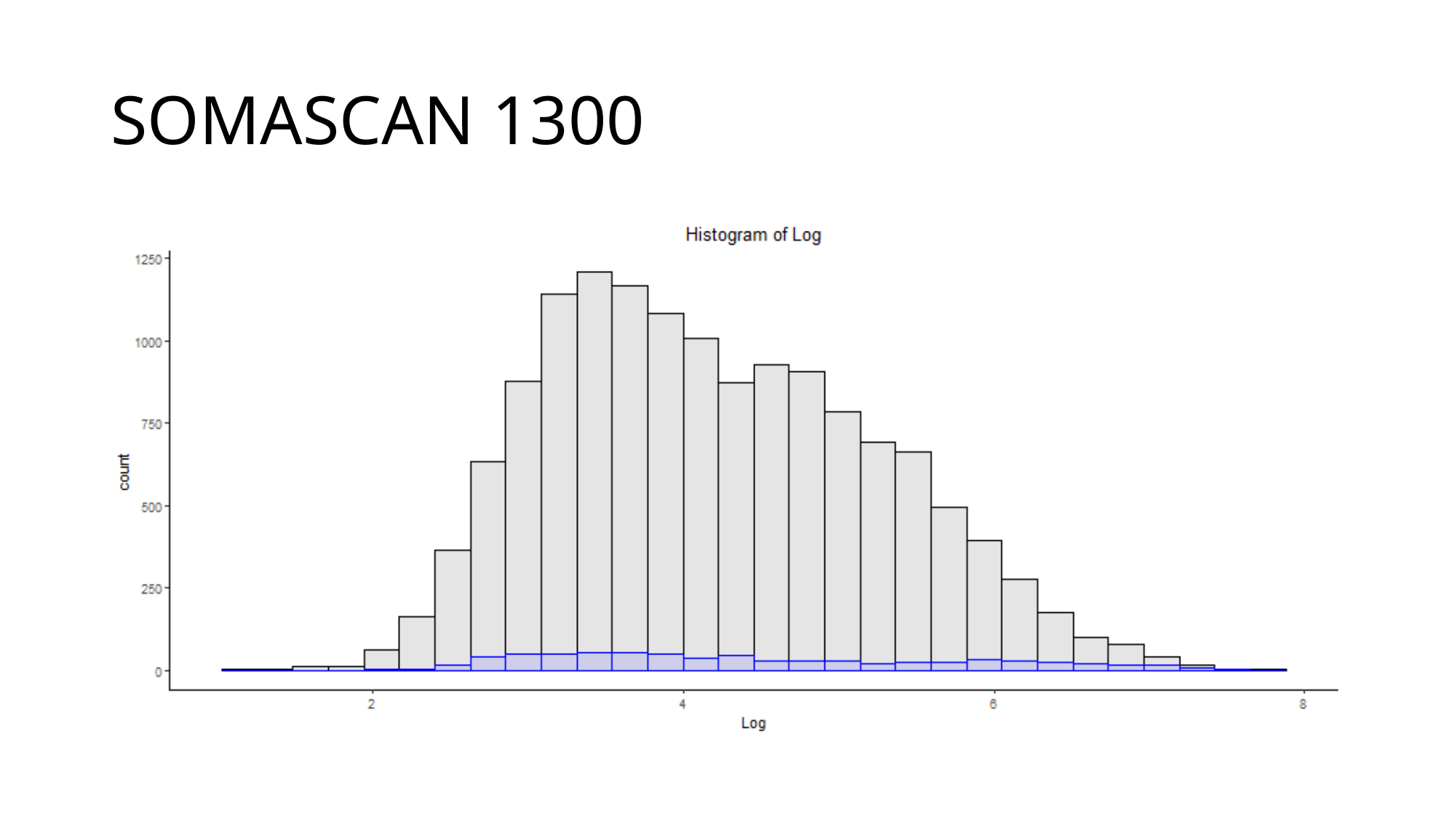

# SOMASCAN 1300

## Slide 3
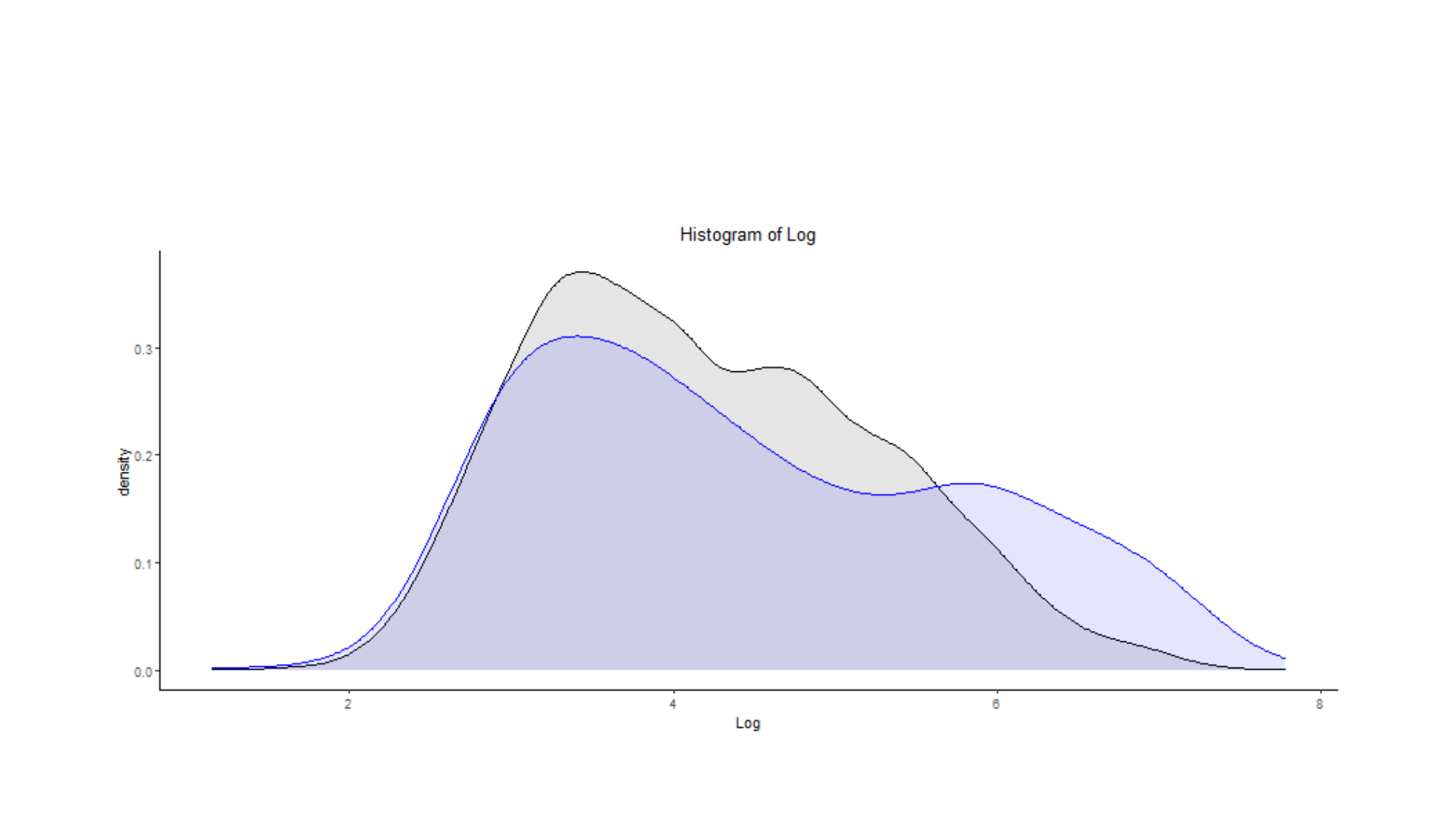

#

## Slide 4
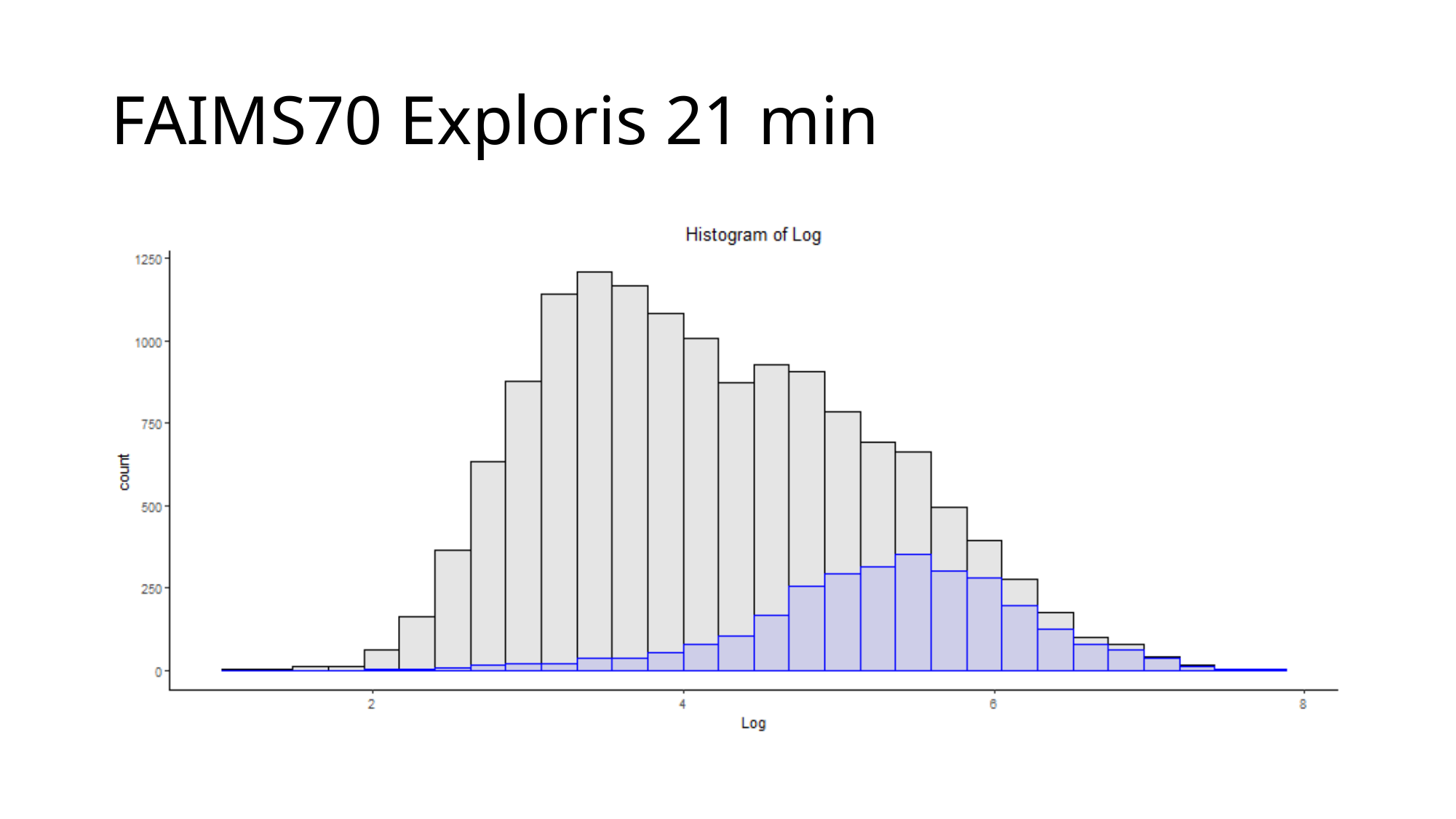

# FAIMS70 Exploris 21 min

## Slide 5
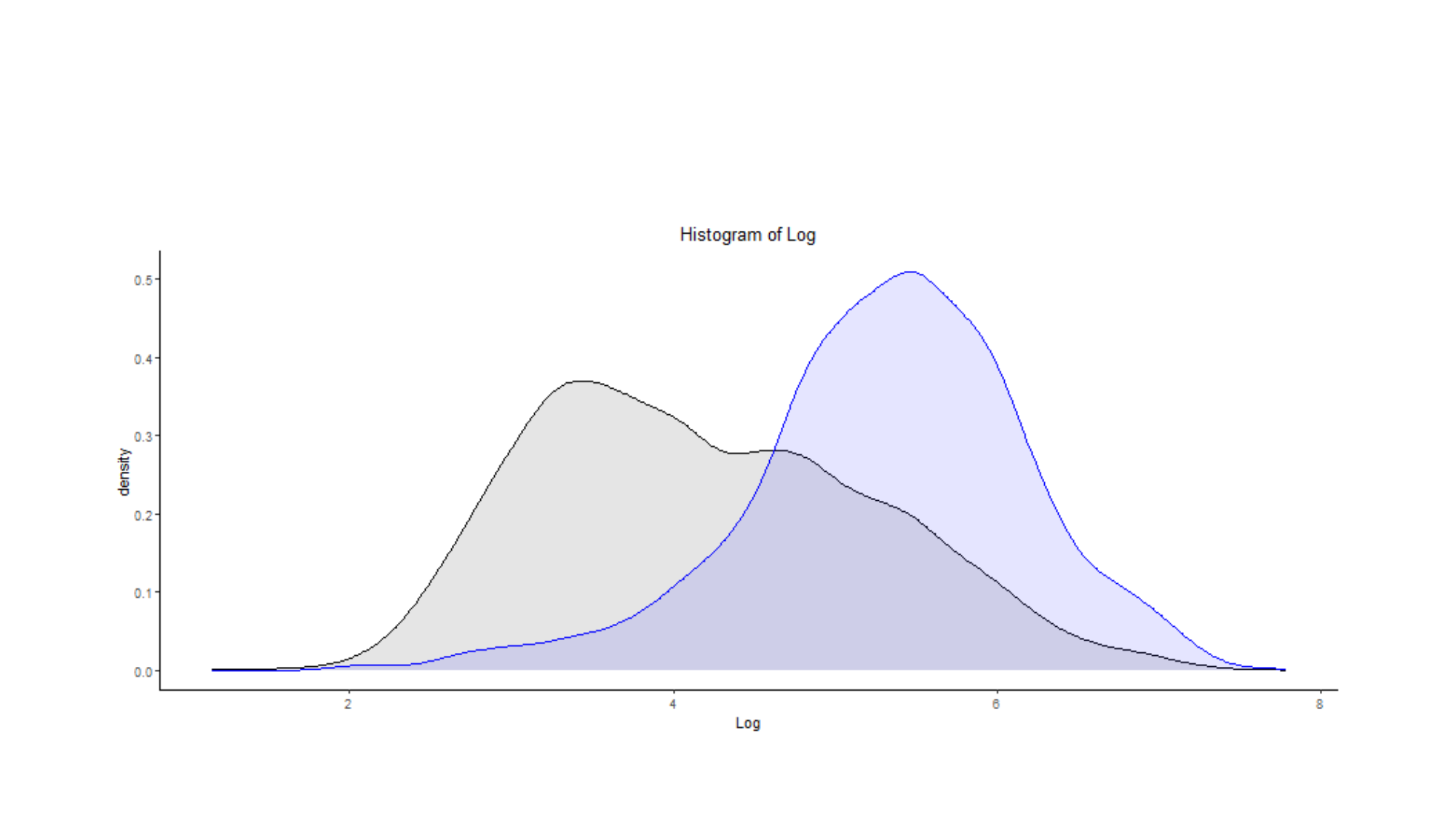

#

## Slide 6
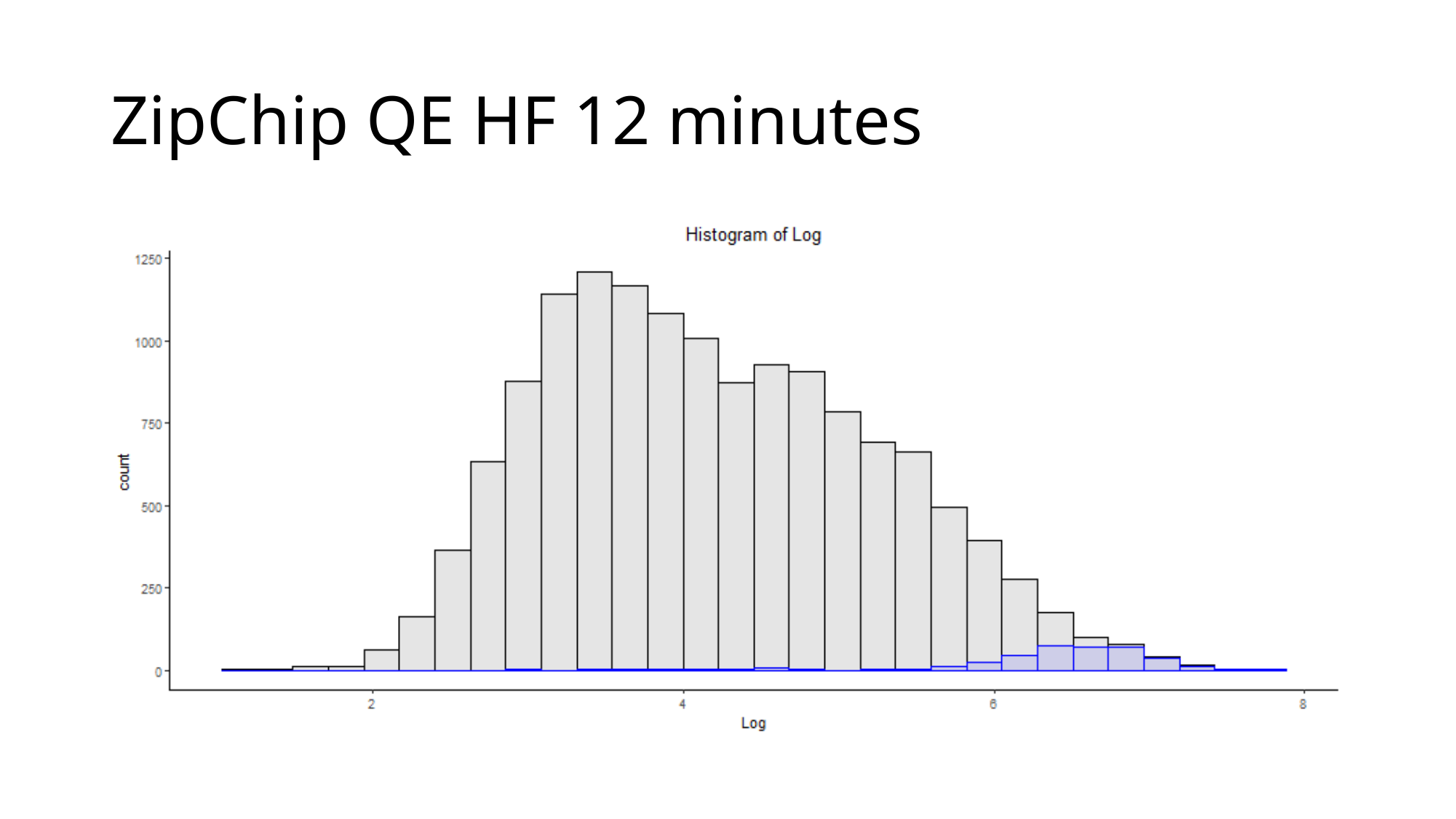

# ZipChip QE HF 12 minutes

## Slide 7
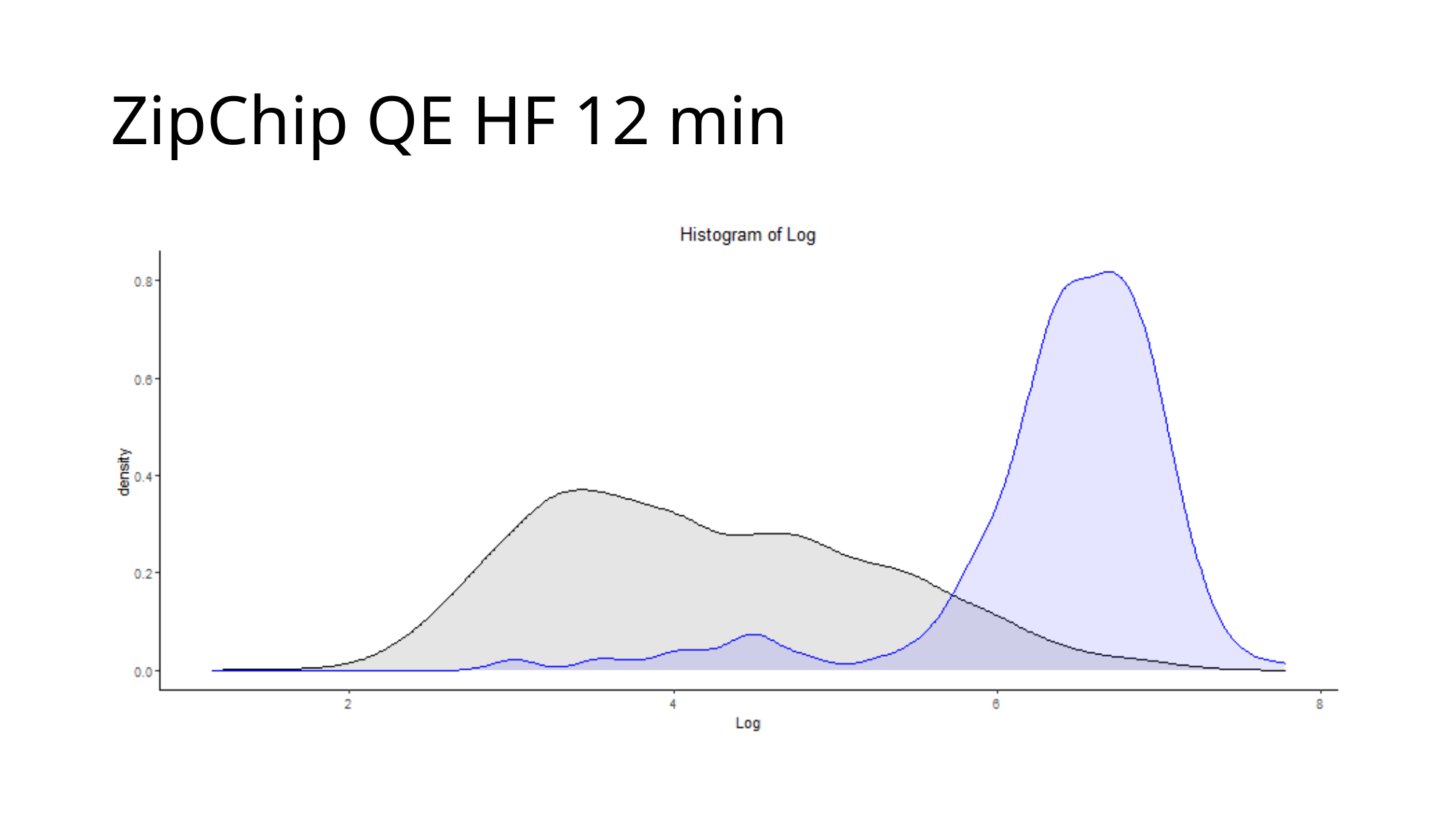

# ZipChip QE HF 12 min

## Slide 8
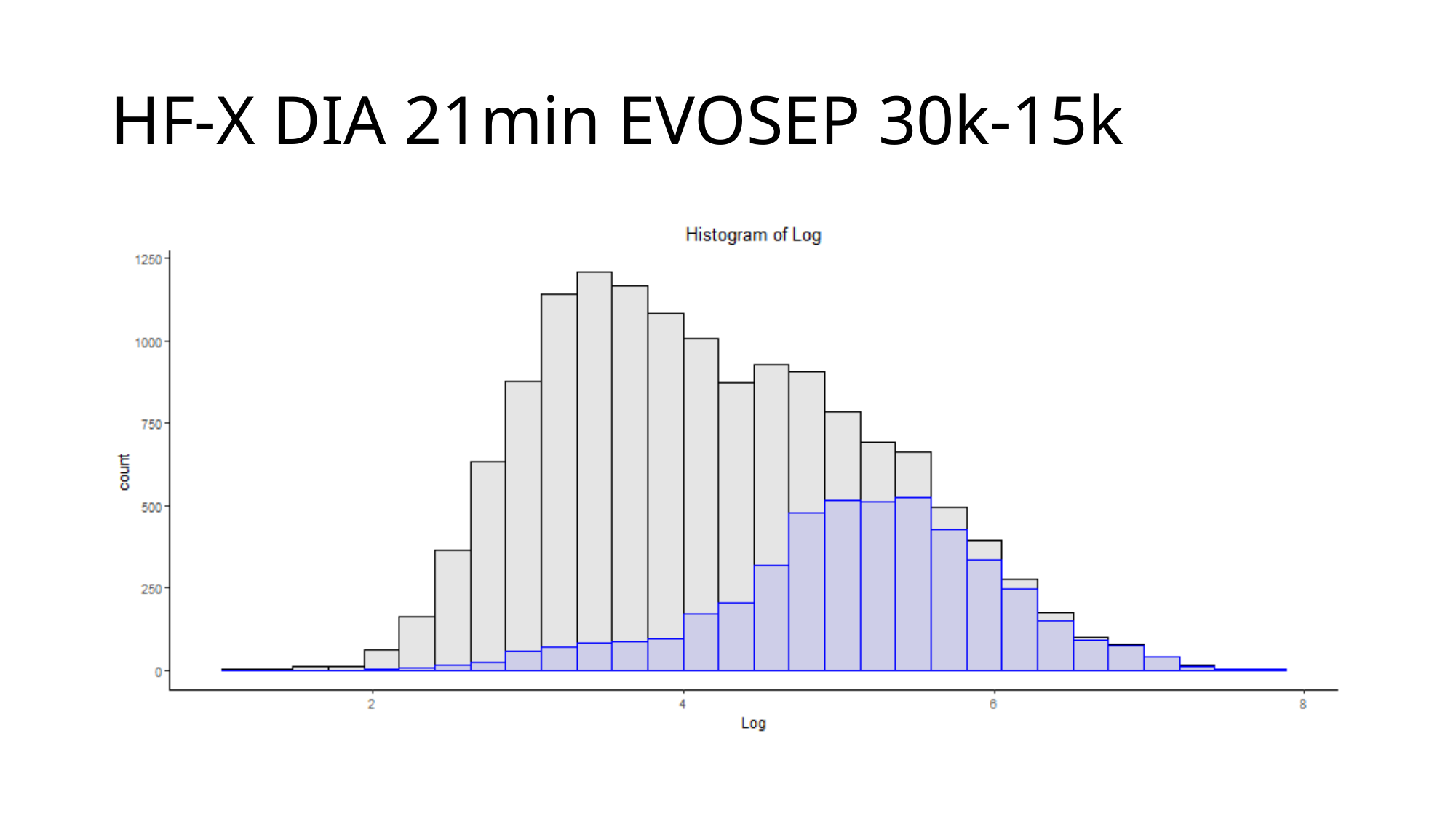

# HF-X DIA 21min EVOSEP 30k-15k

## Slide 9
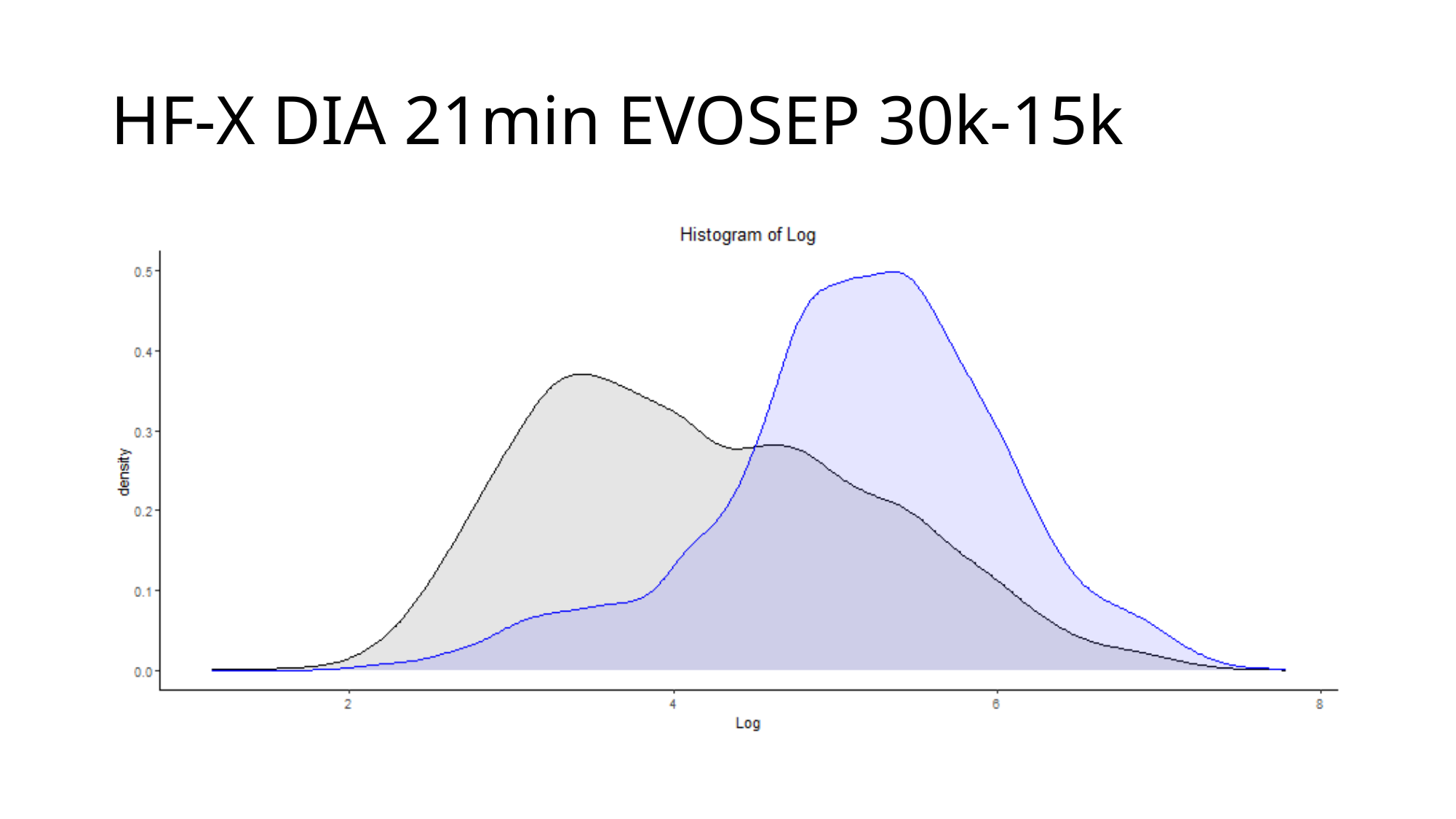

# HF-X DIA 21min EVOSEP 30k-15k

## Slide 10
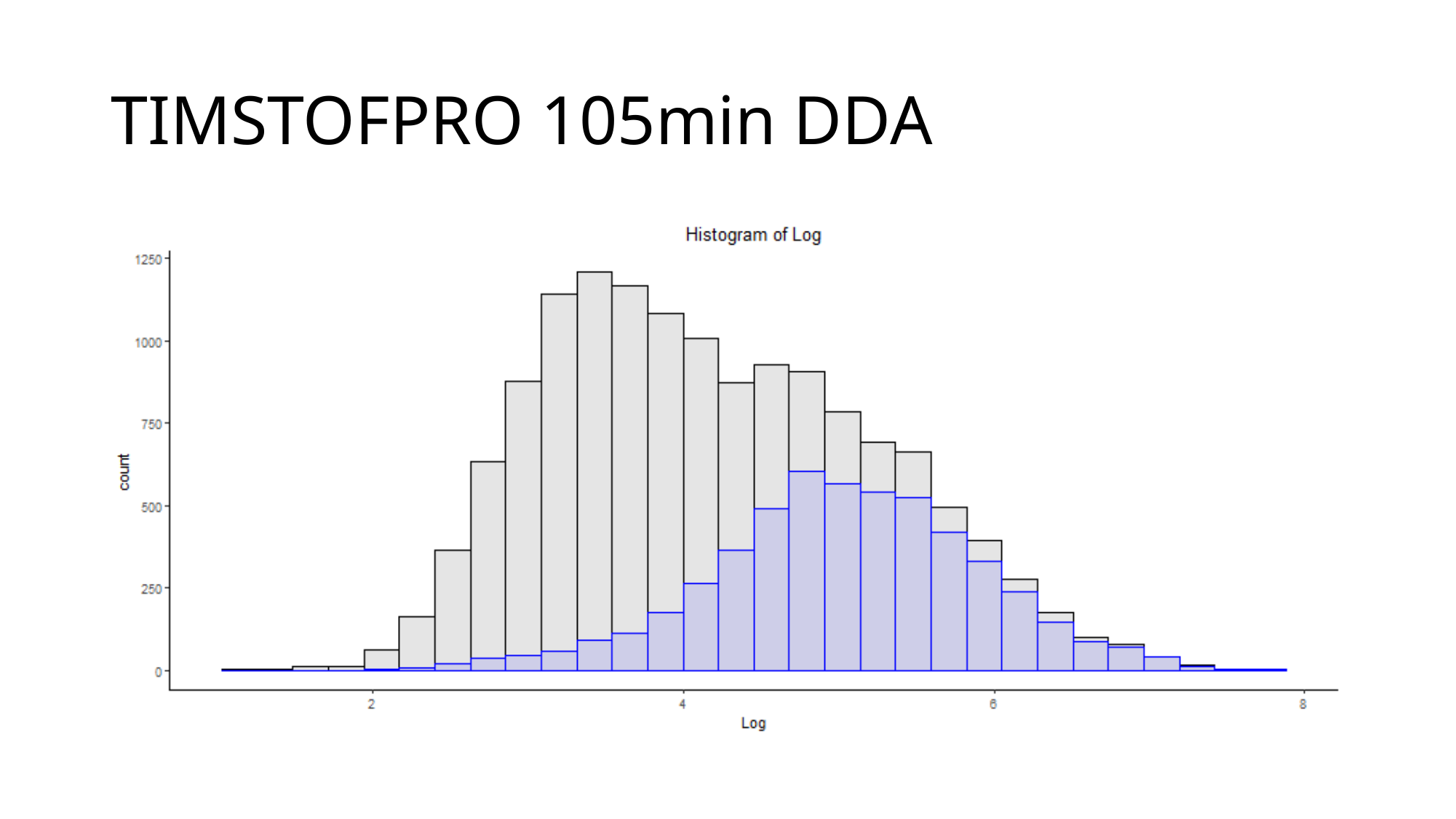

# TIMSTOFPRO 105min DDA

## Slide 11
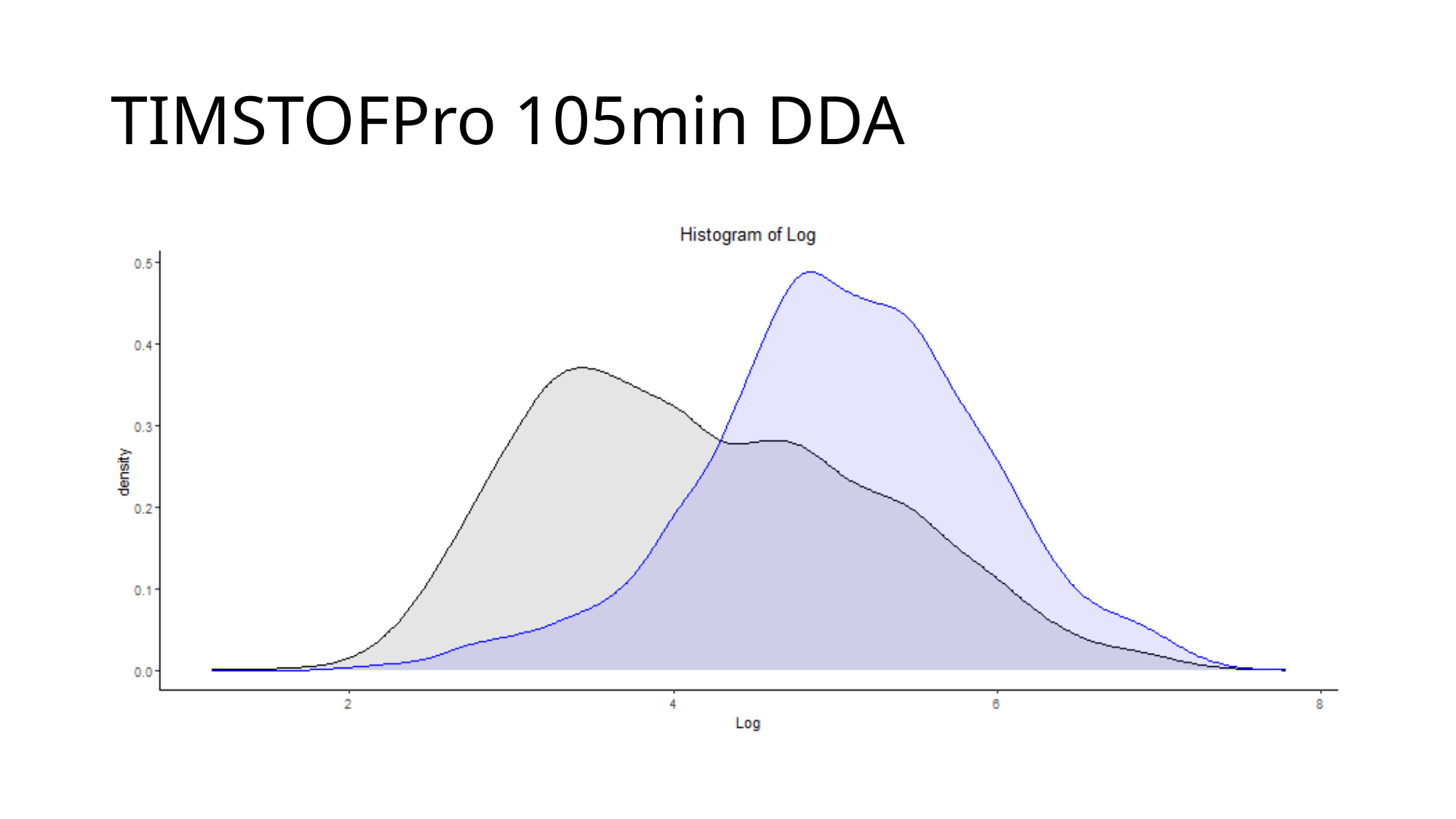

# TIMSTOFPro 105min DDA

## Slide 12
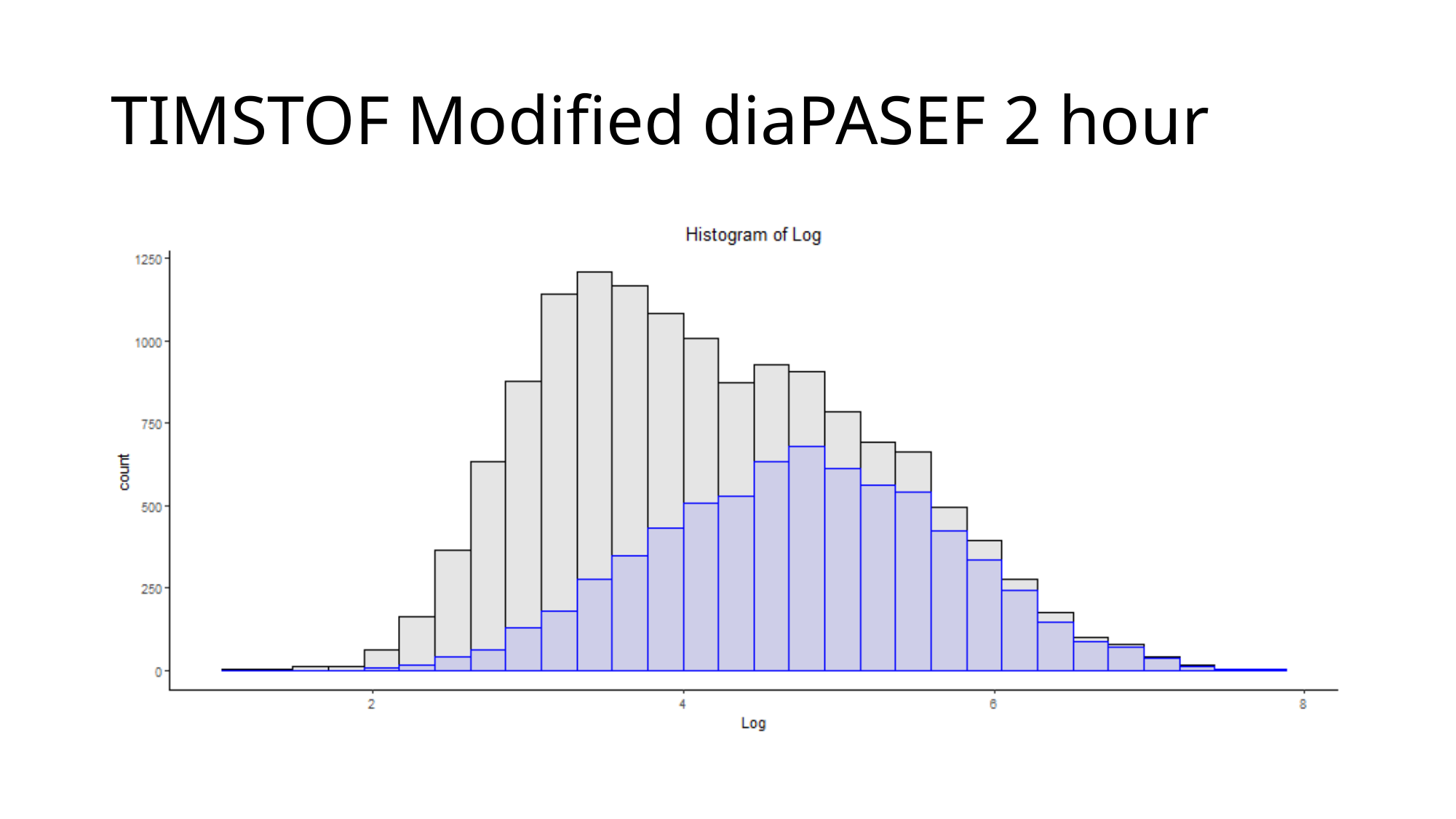

# TIMSTOF Modified diaPASEF 2 hour

## Slide 13
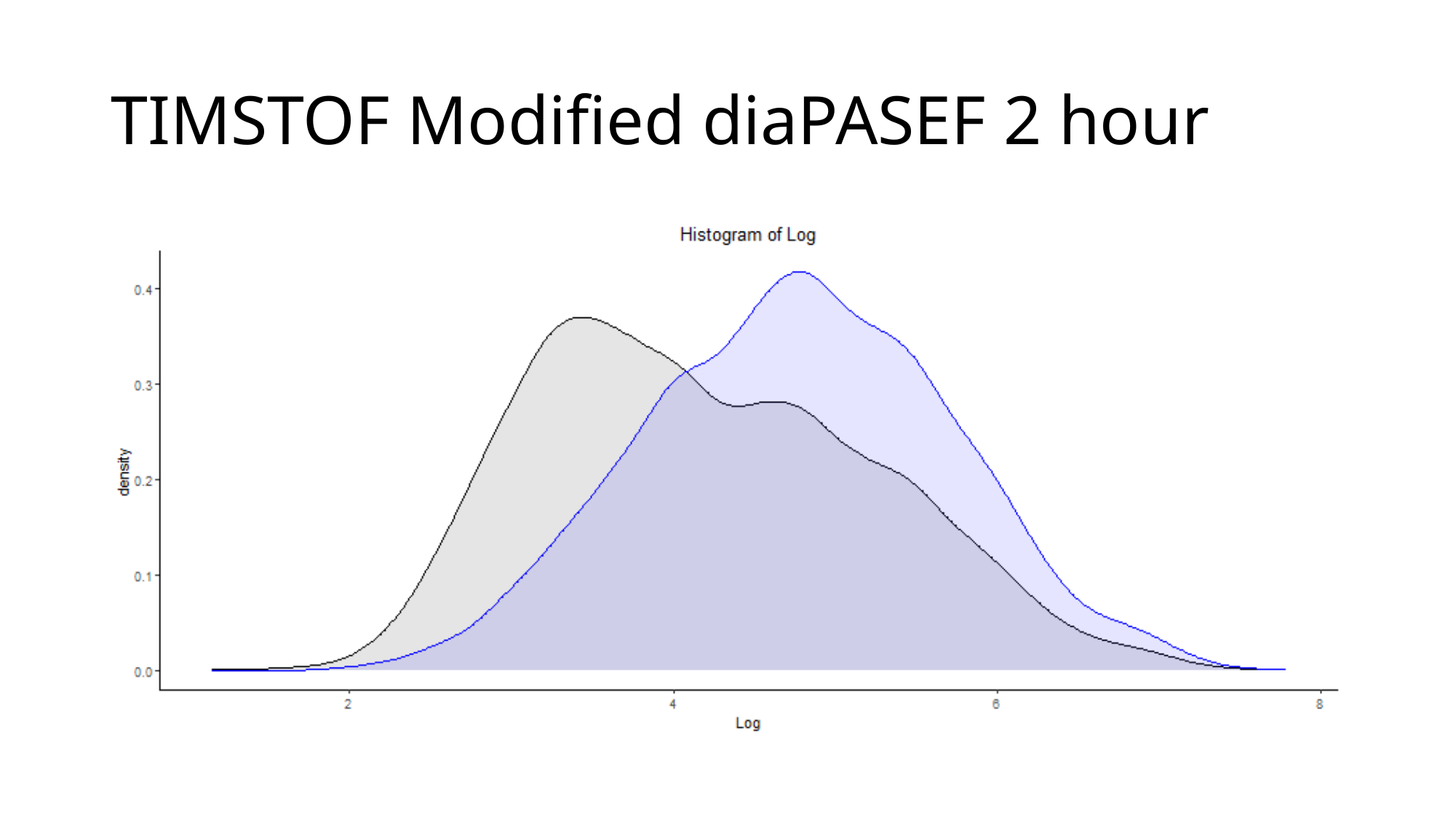

# TIMSTOF Modified diaPASEF 2 hour

## Slide 14
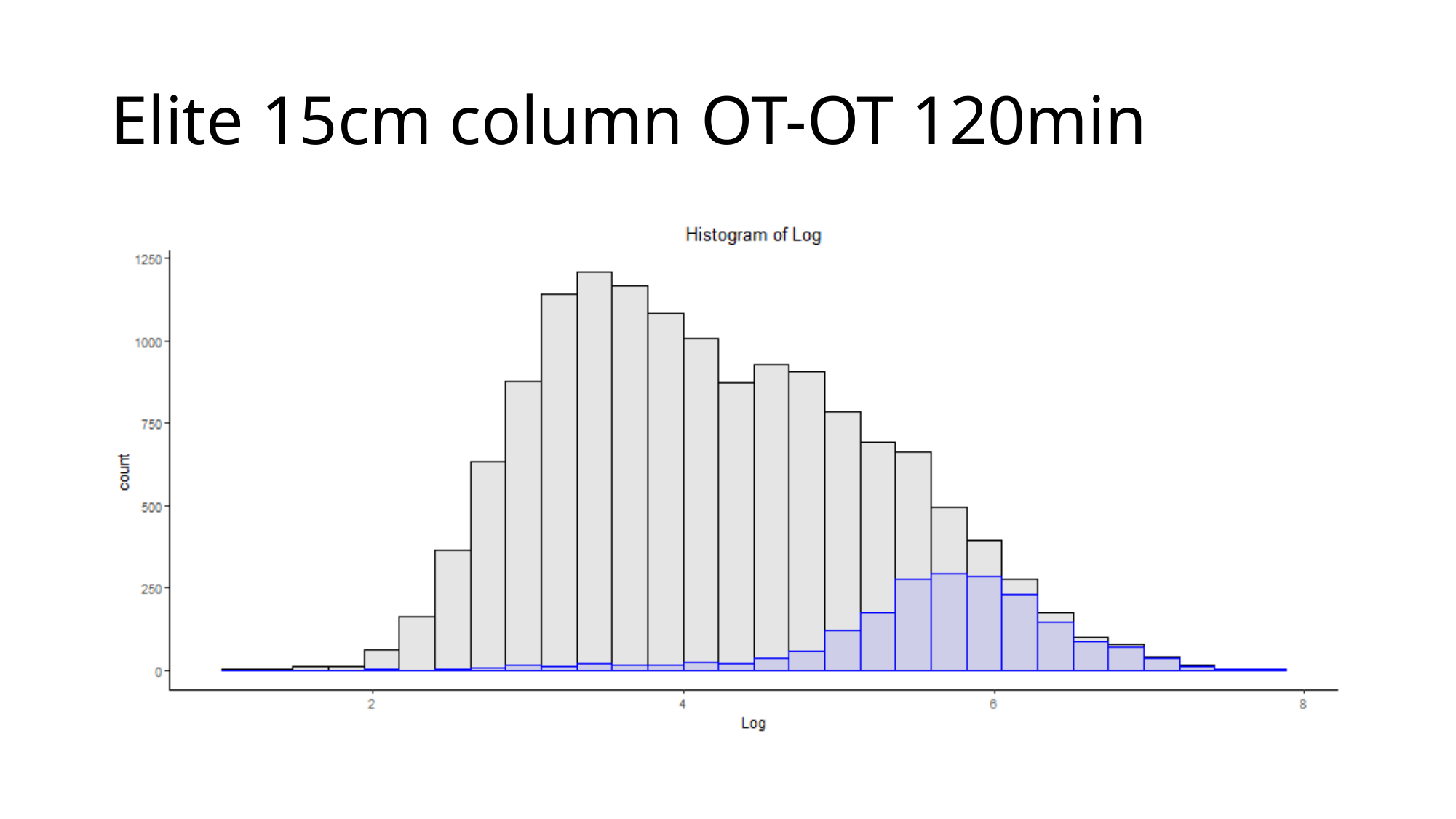

# Elite 15cm column OT-OT 120min

## Slide 15
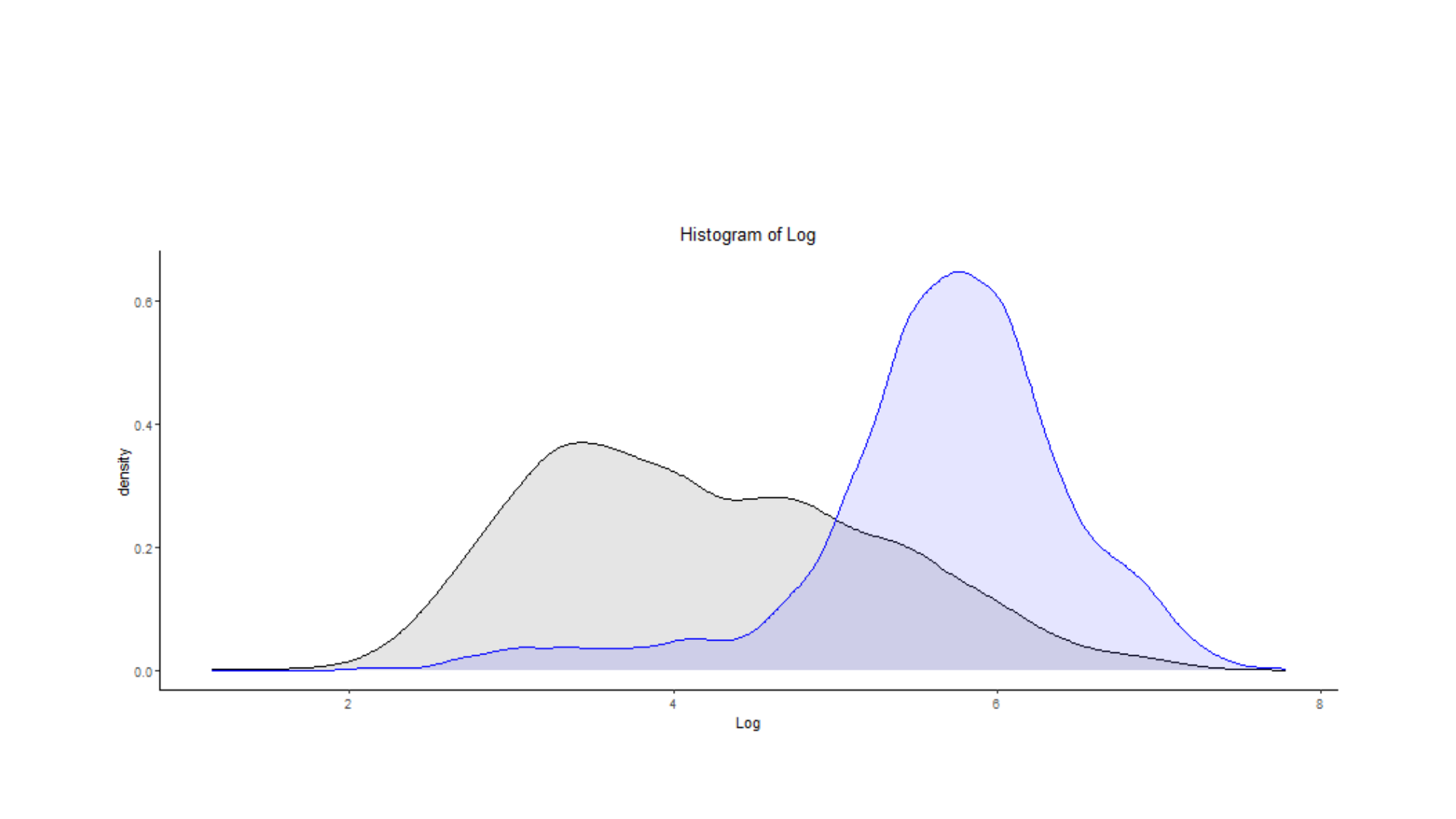

#

## Slide 16
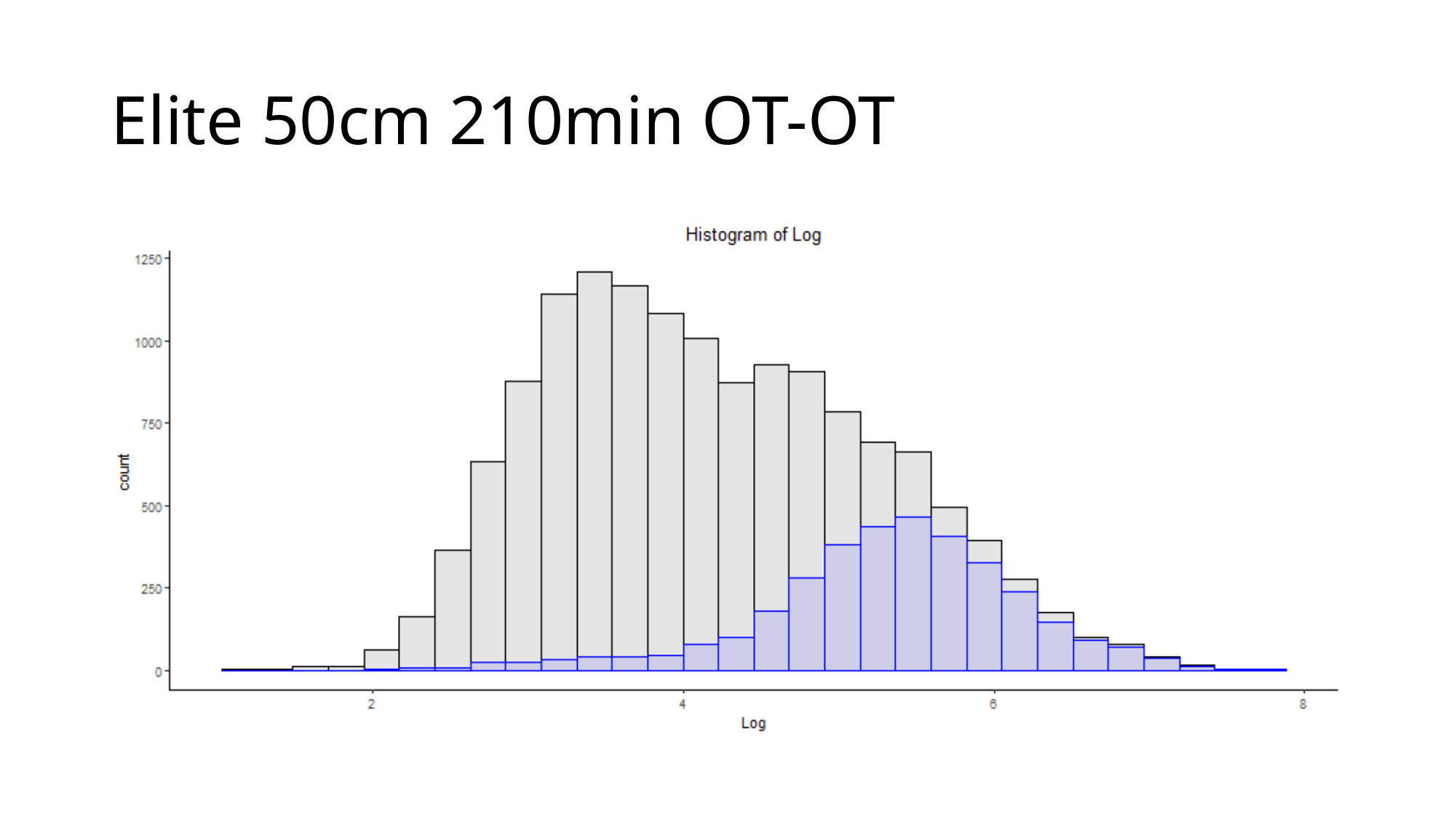

# Elite 50cm 210min OT-OT

## Slide 17
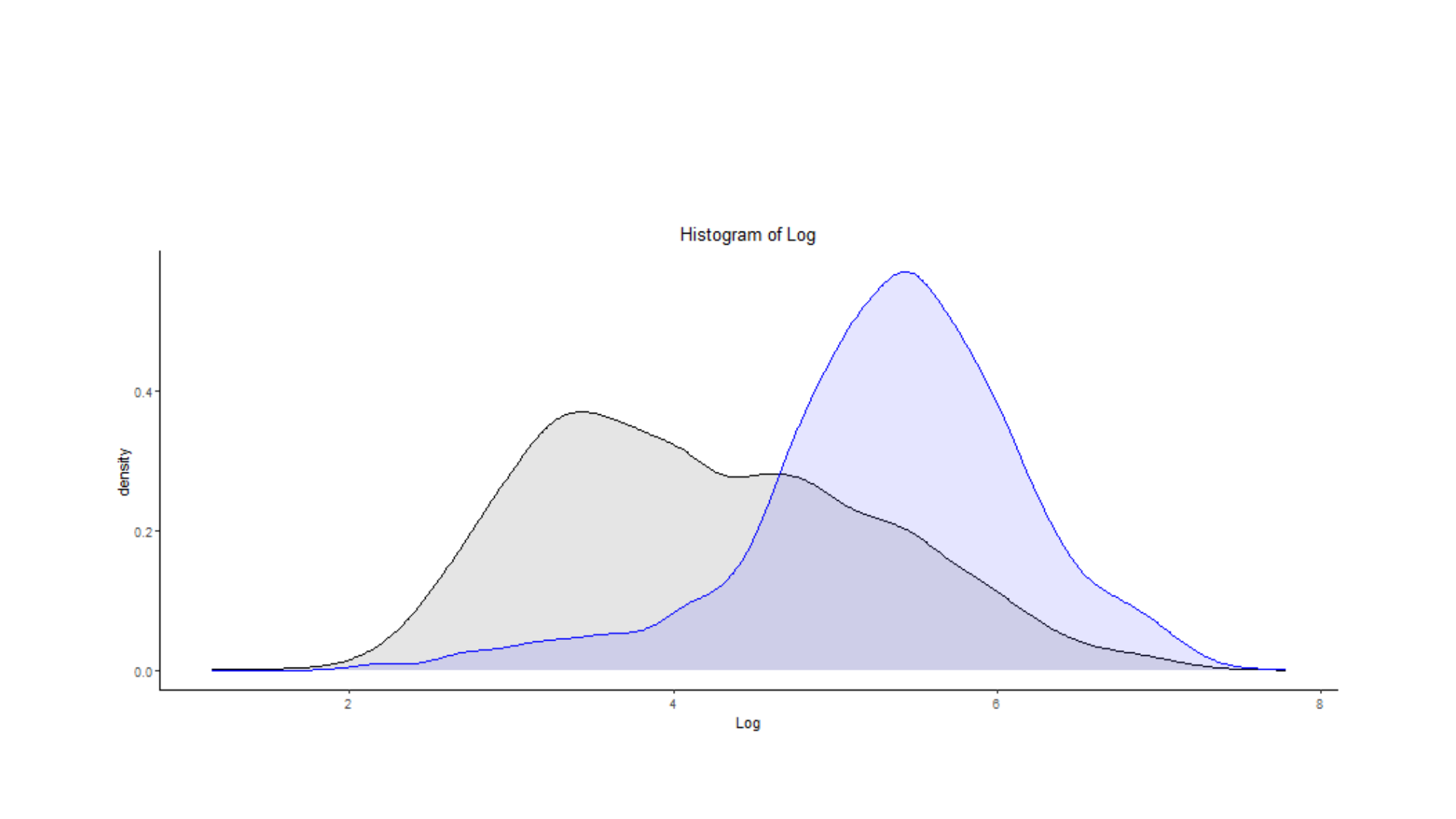

#

## Slide 18
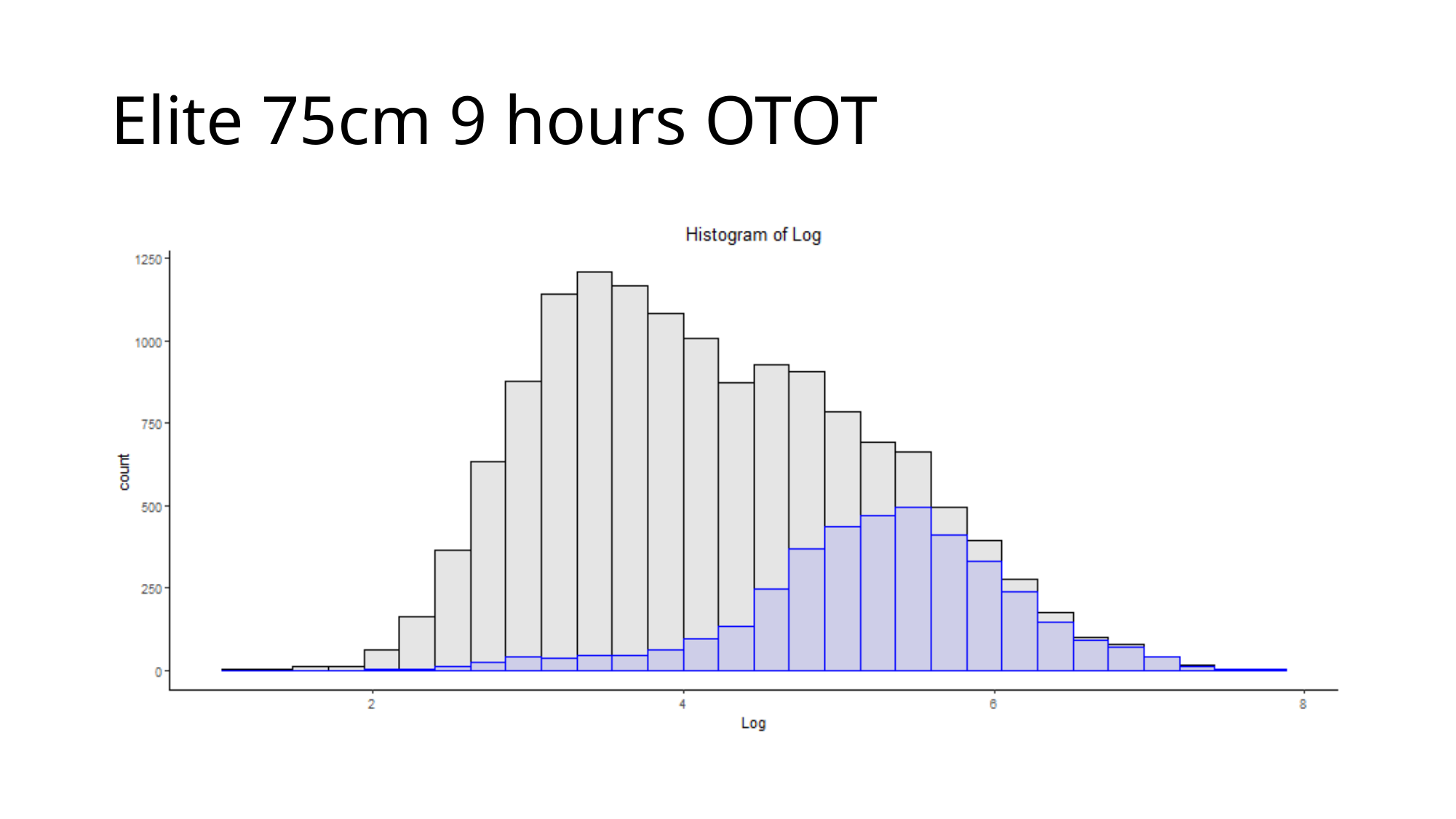

# Elite 75cm 9 hours OTOT

## Slide 19
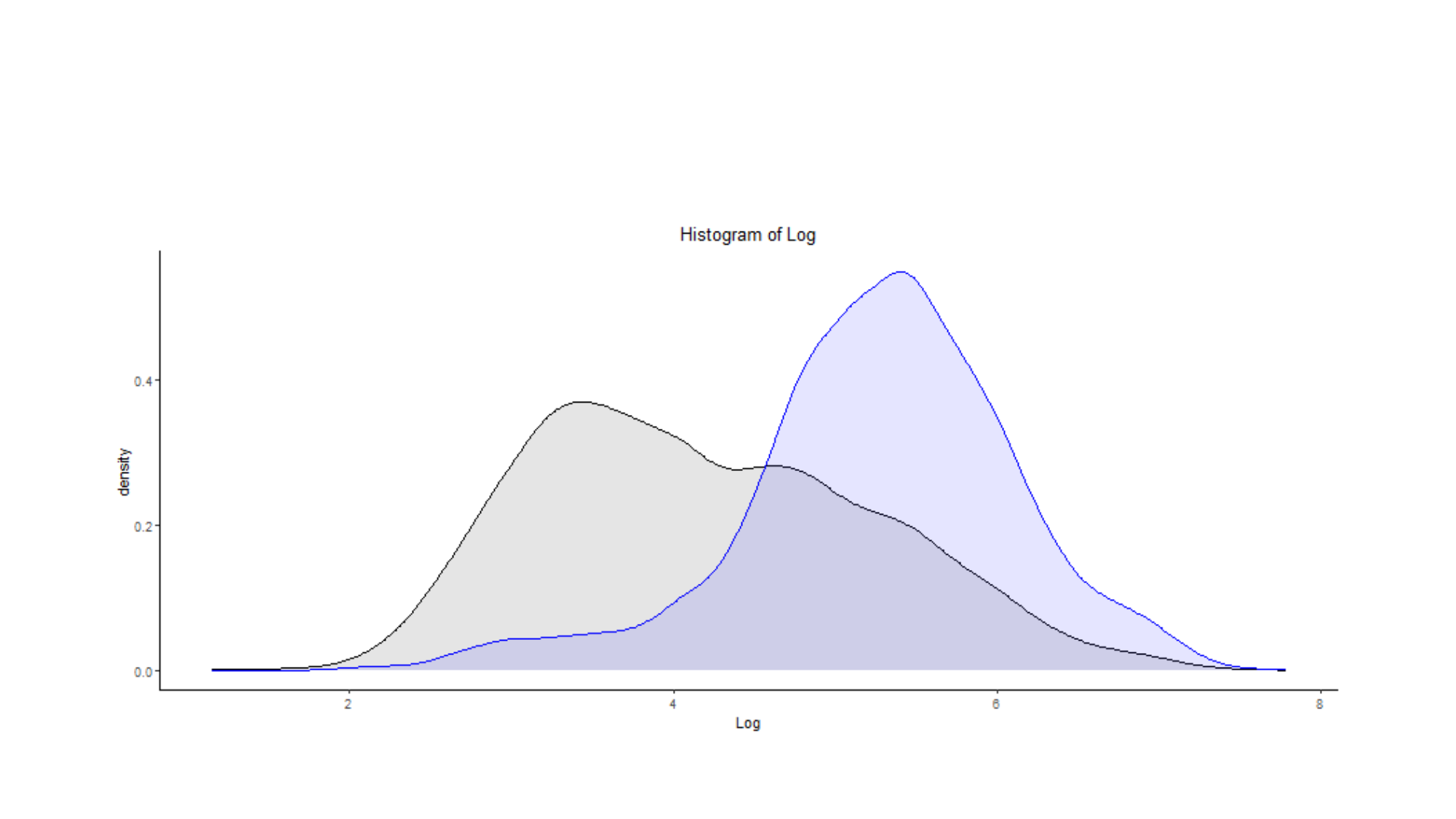

#

## Slide 20
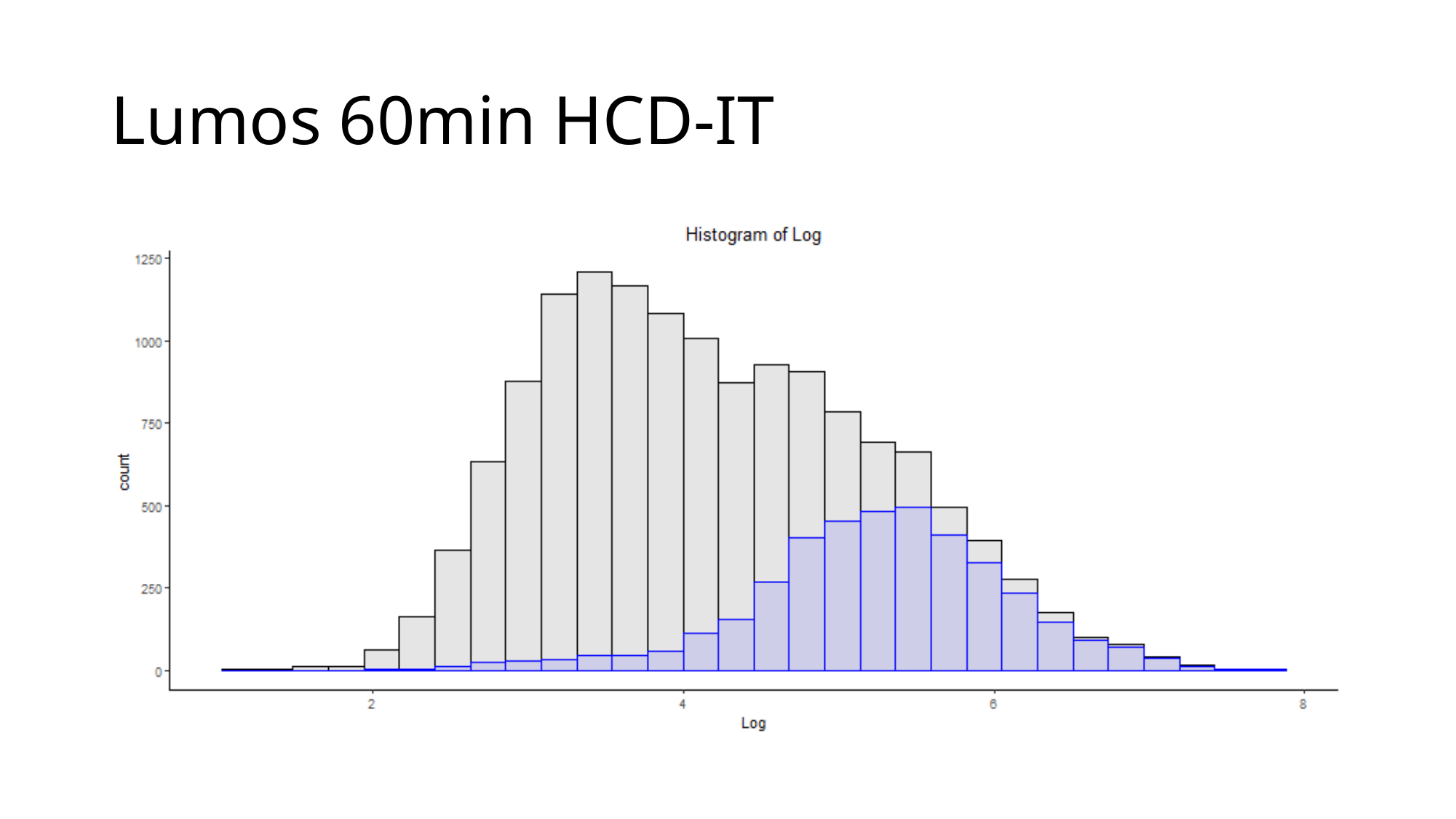

# Lumos 60min HCD-IT

## Slide 21
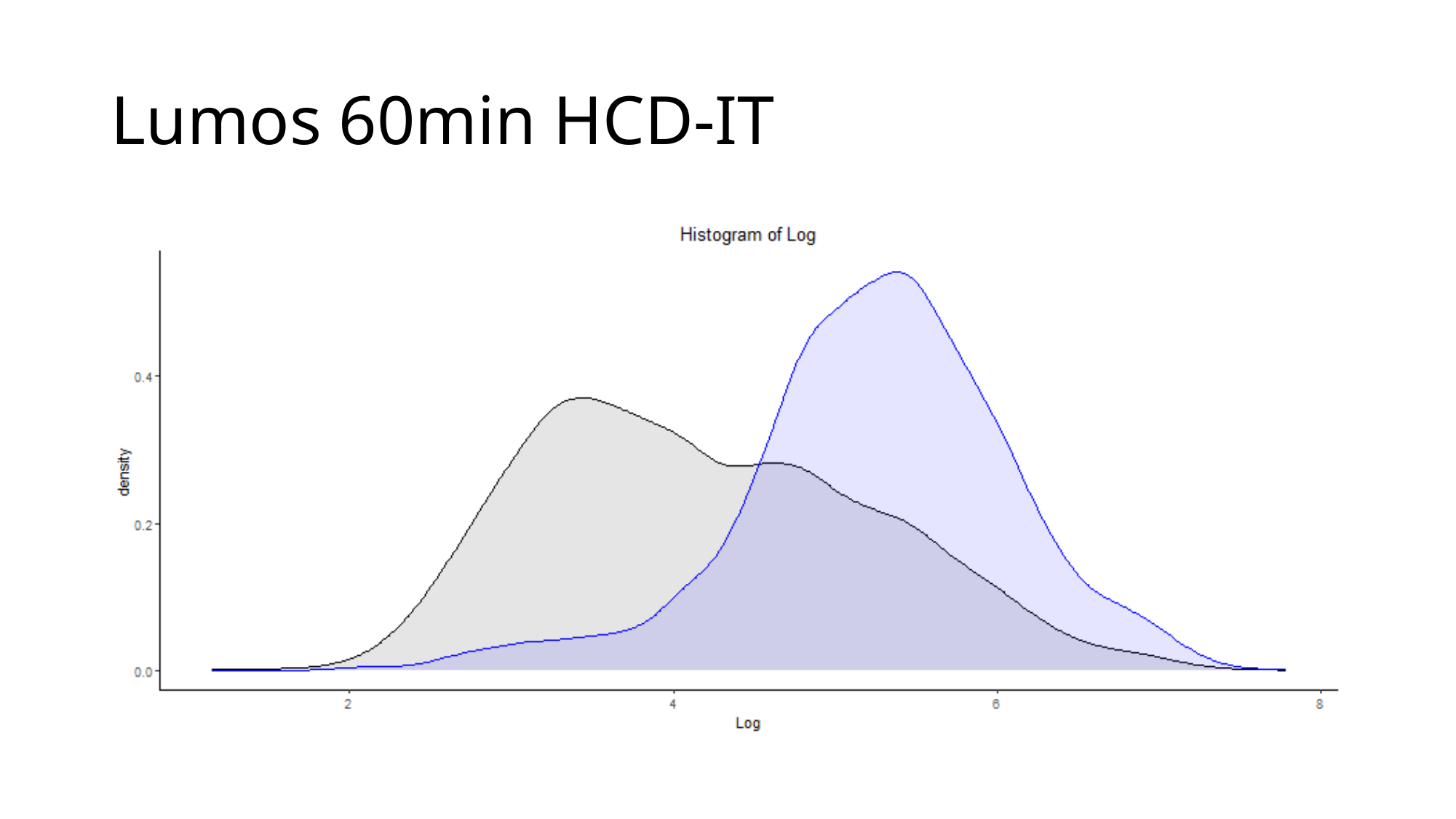

# Lumos 60min HCD-IT

## Slide 22
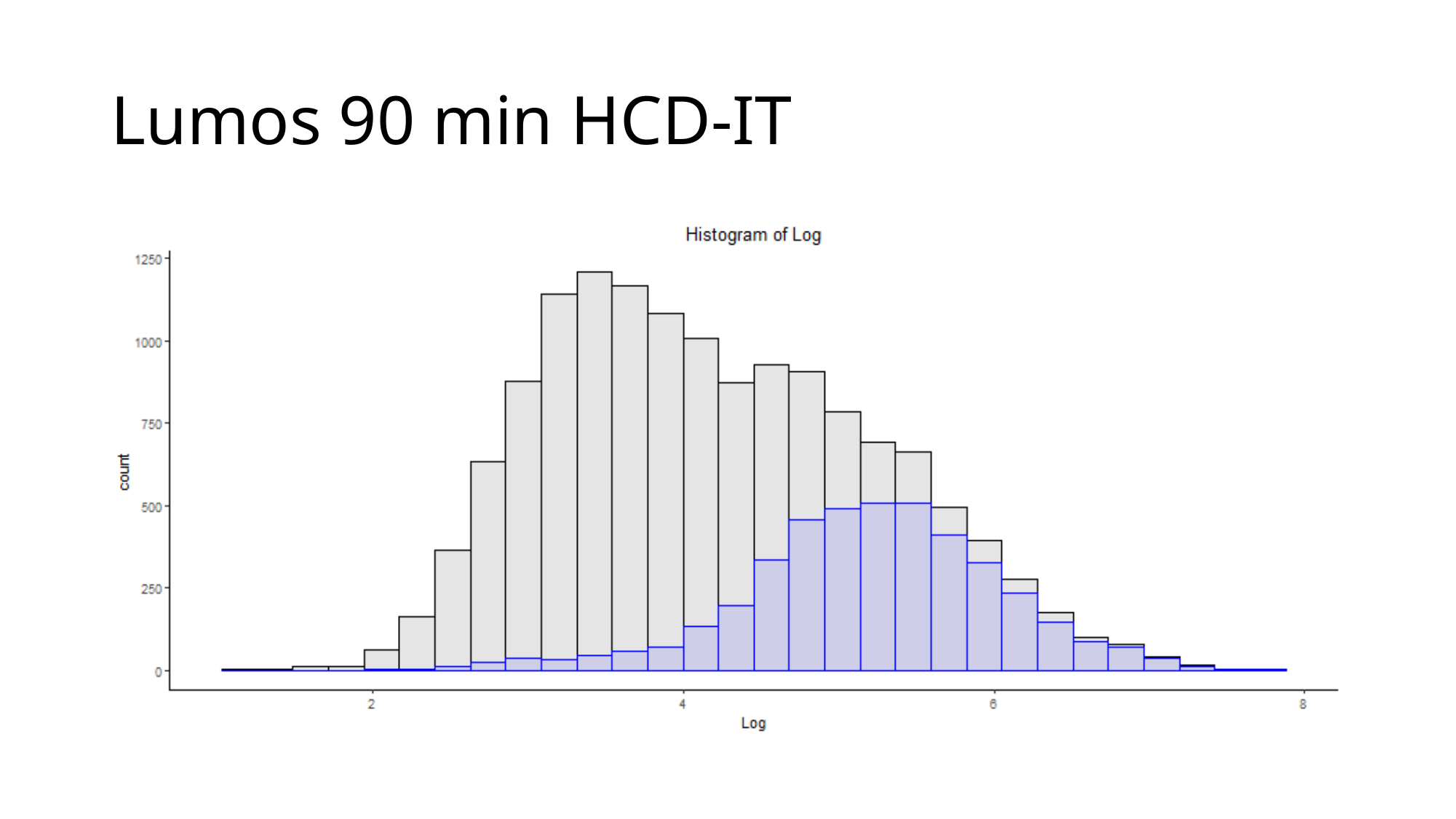

# Lumos 90 min HCD-IT

## Slide 23
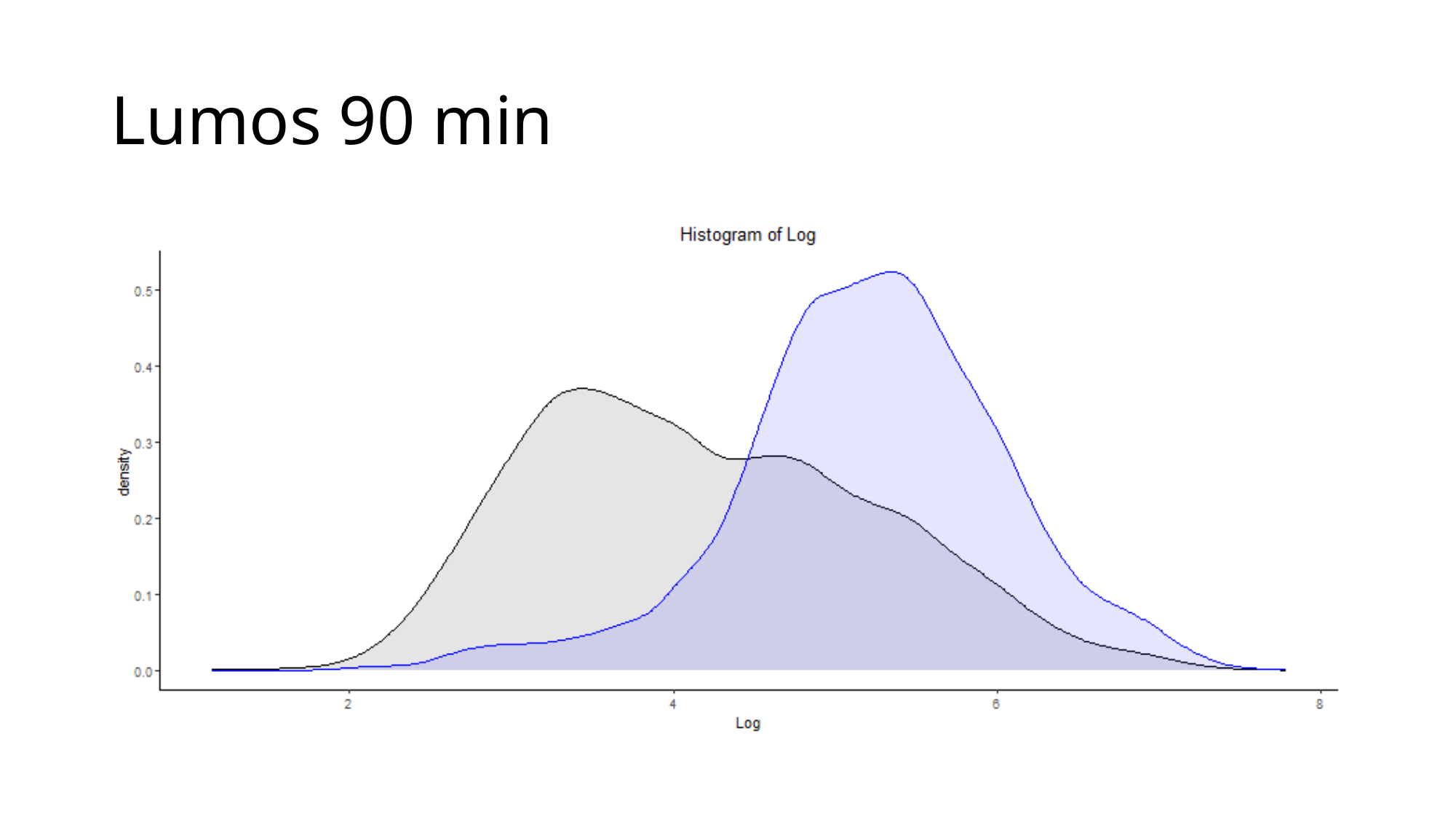

# Lumos 90 min

## Slide 24
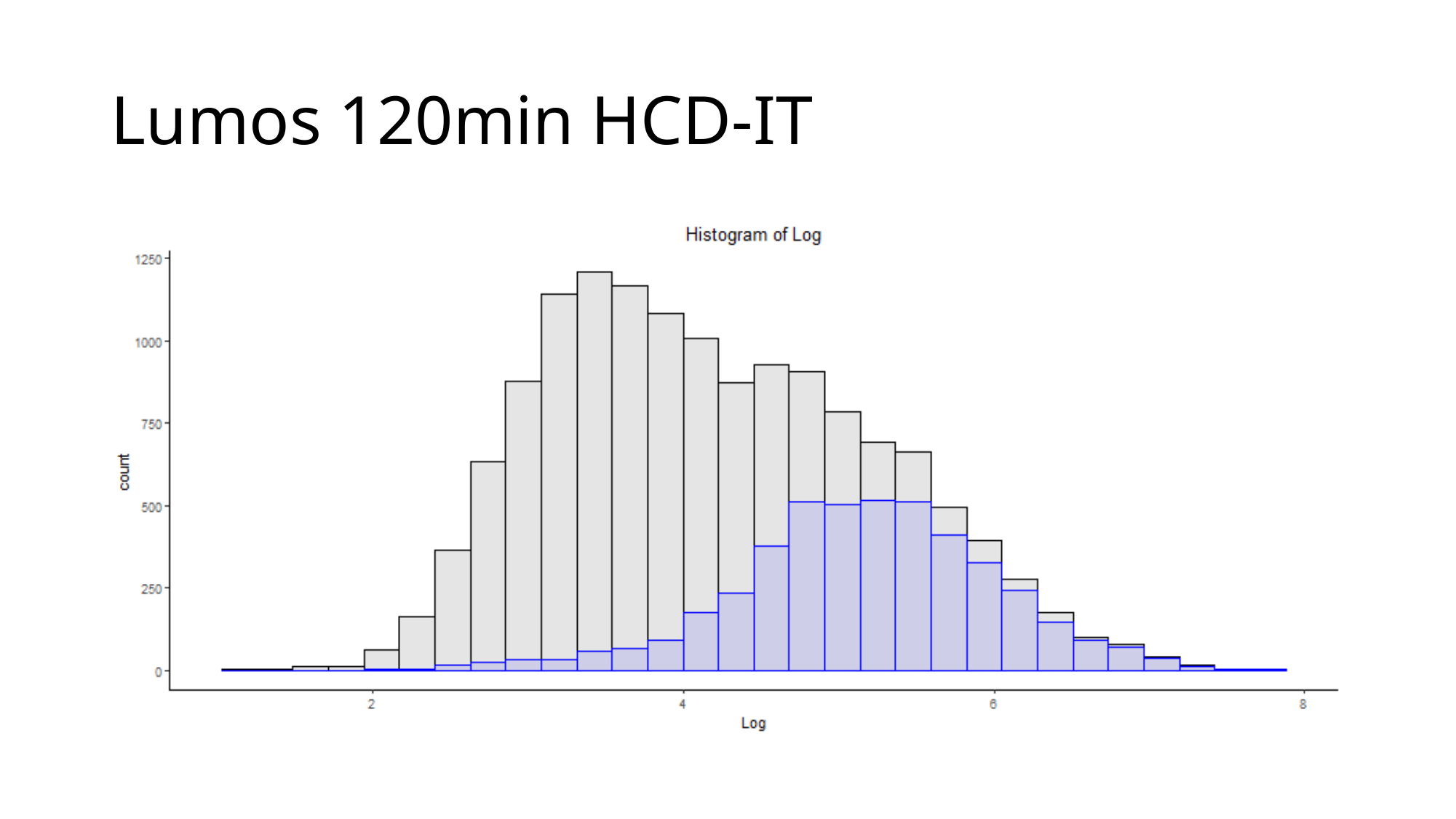

# Lumos 120min HCD-IT

## Slide 25
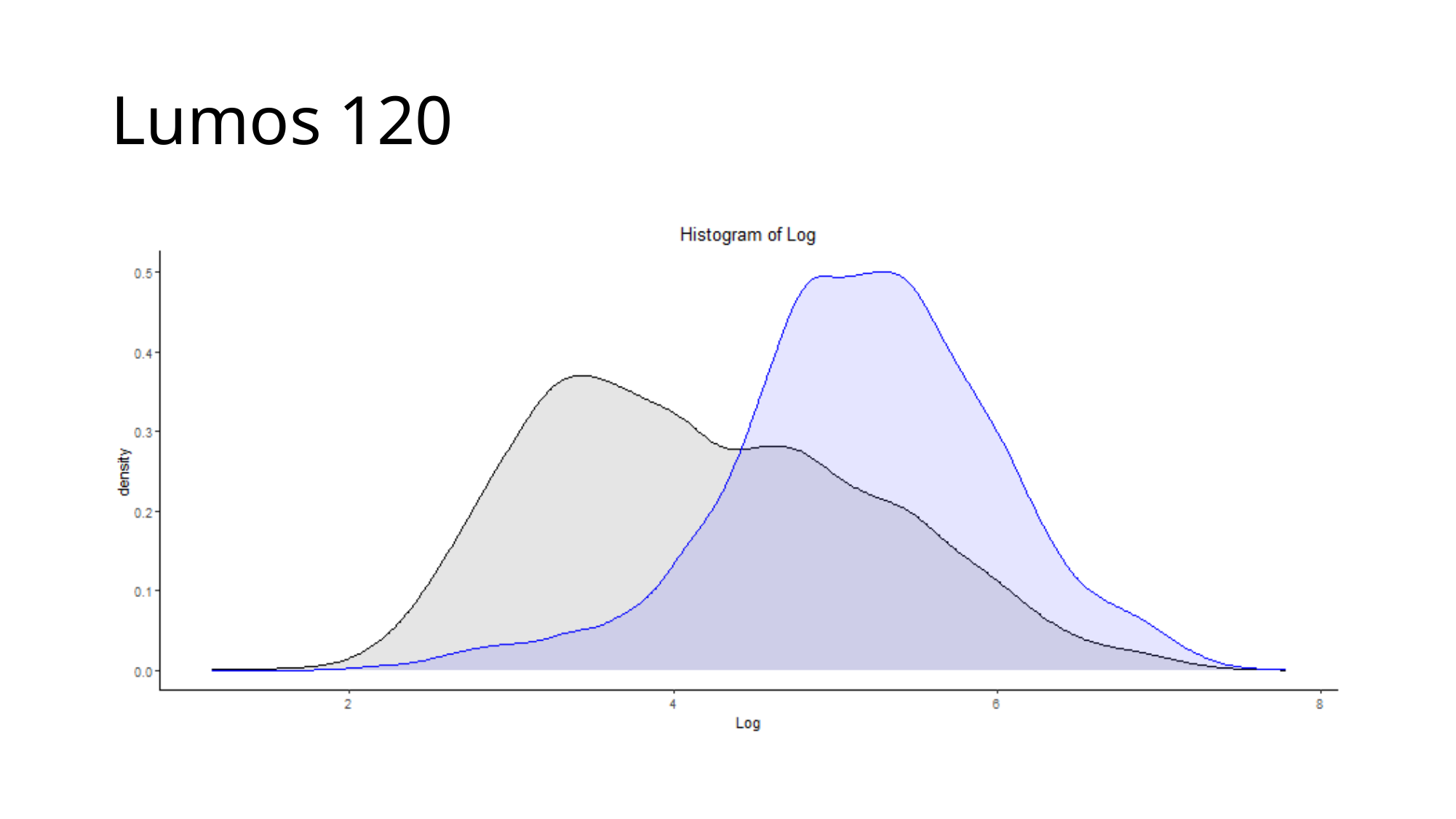

# Lumos 120

## Slide 26
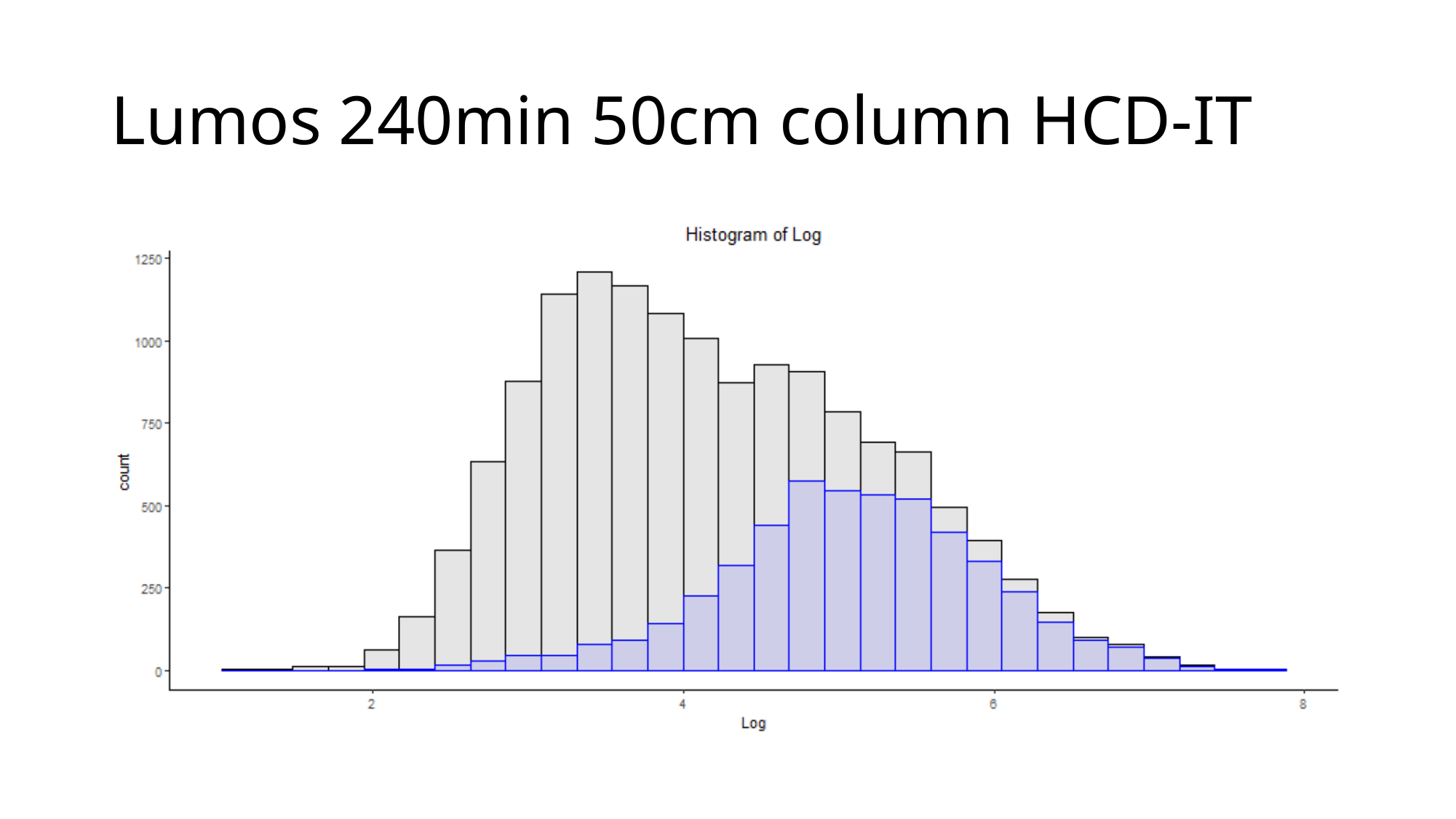

# Lumos 240min 50cm column HCD-IT

## Slide 27
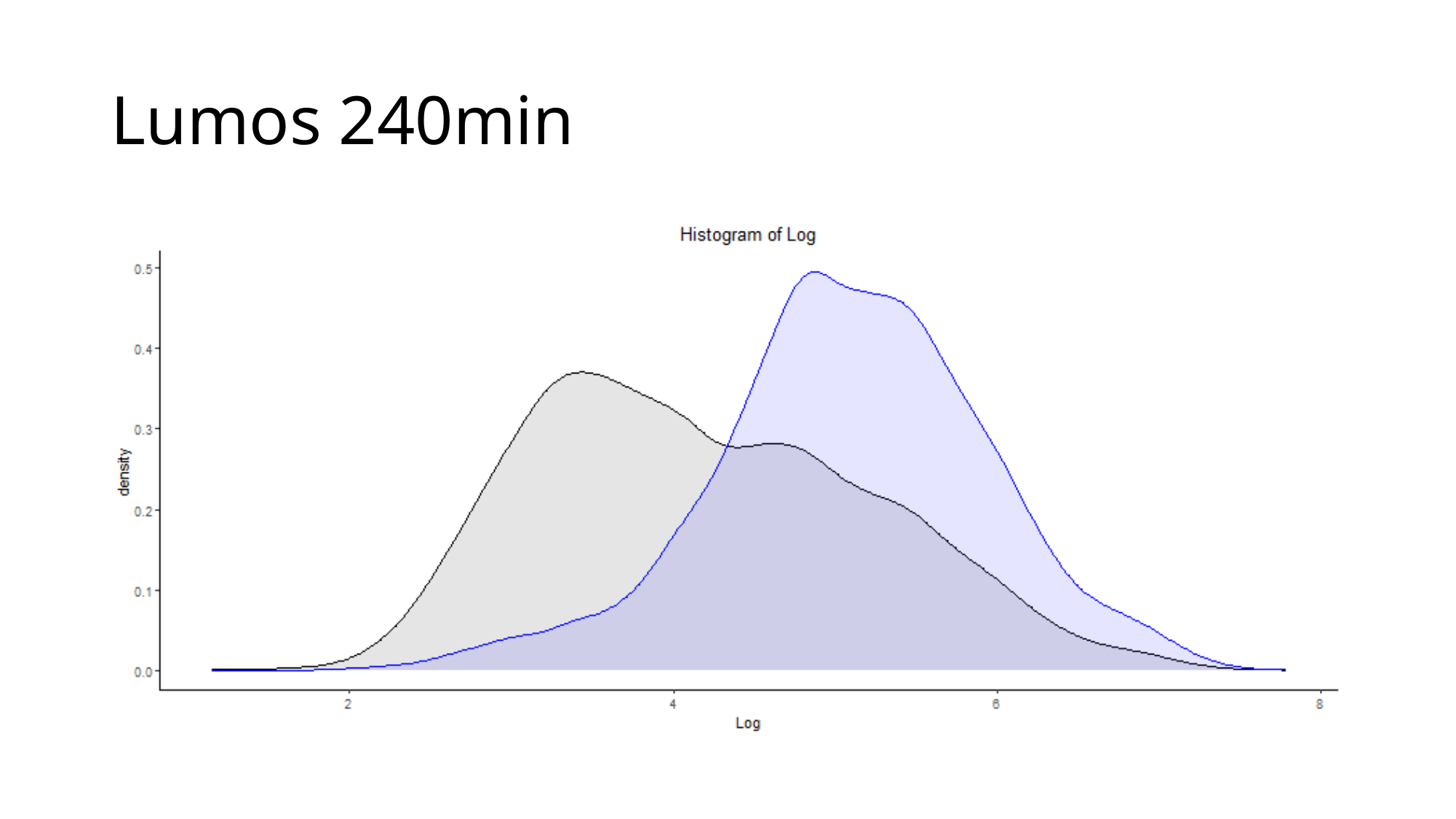

# Lumos 240min

## Slide 28
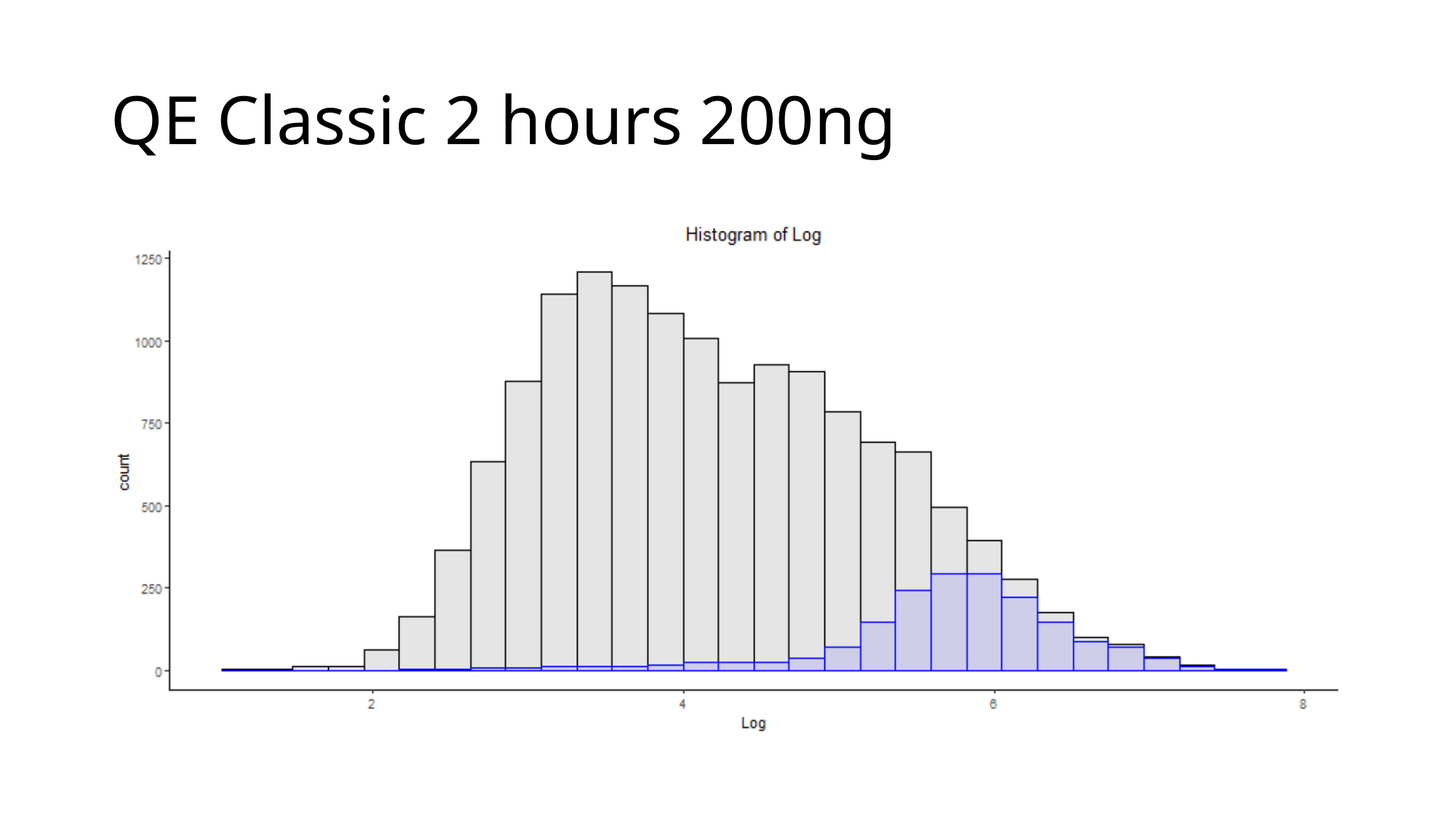

# QE Classic 2 hours 200ng

## Slide 29
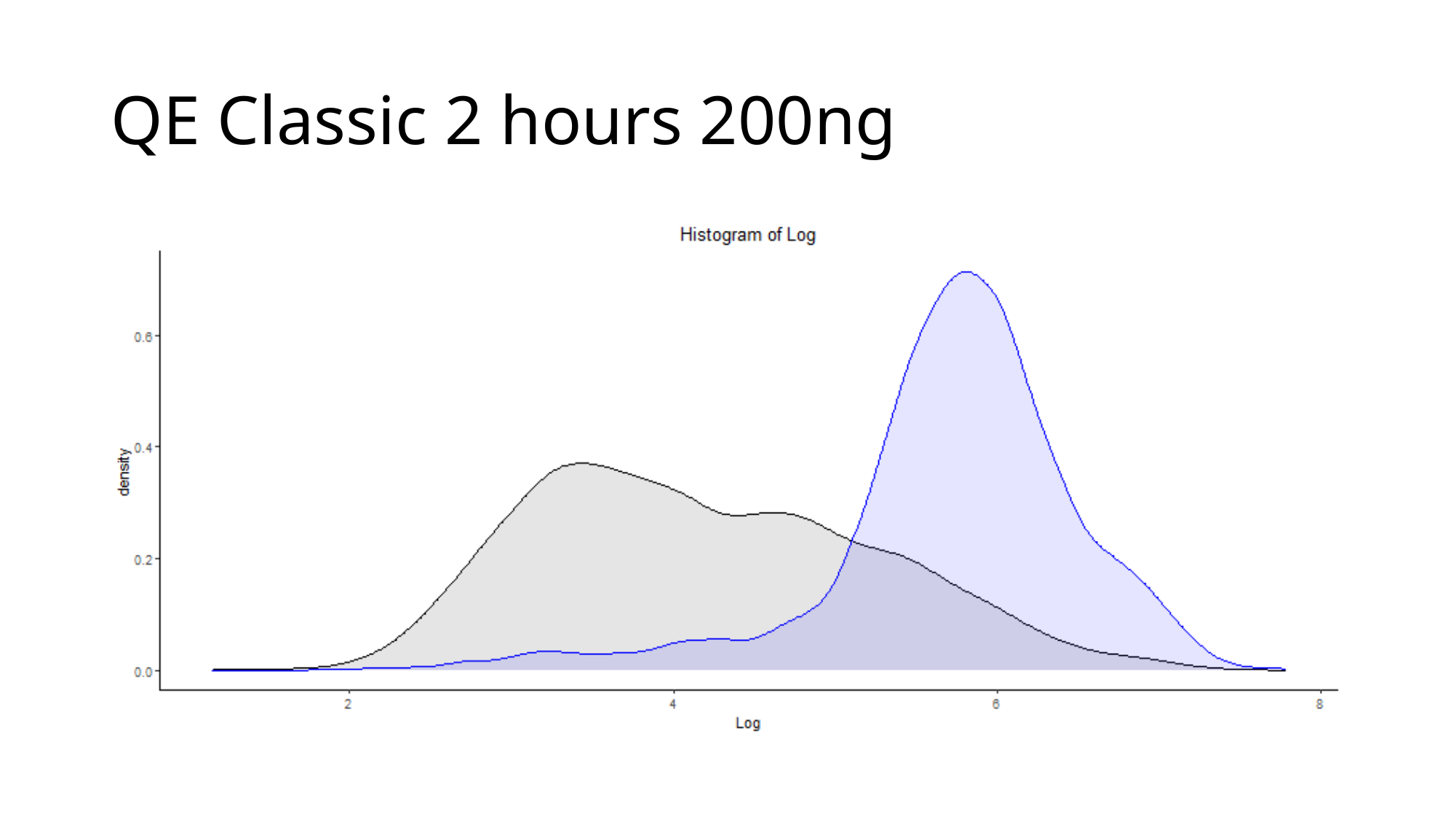

# QE Classic 2 hours 200ng

## Slide 30
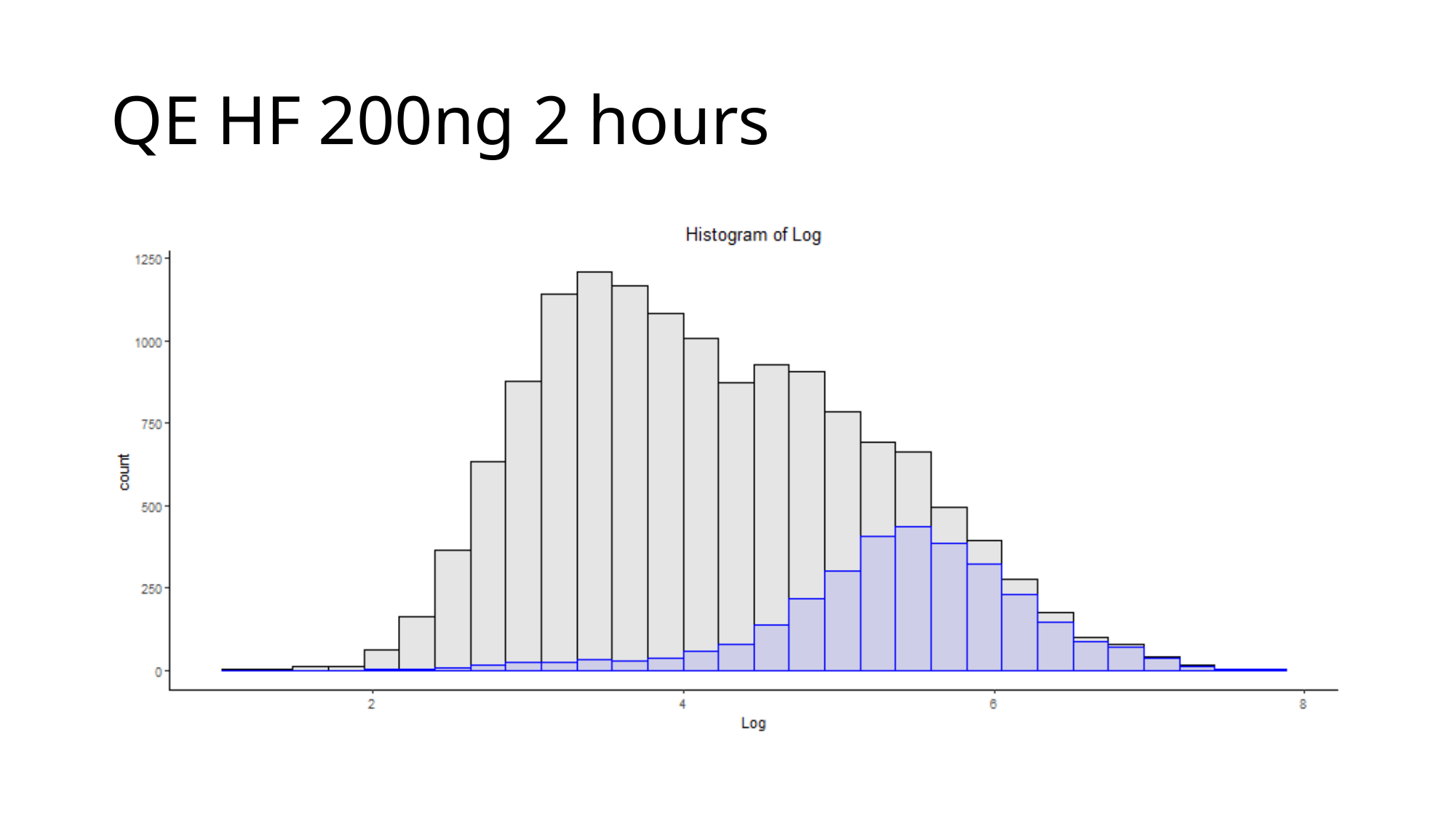

# QE HF 200ng 2 hours

## Slide 31
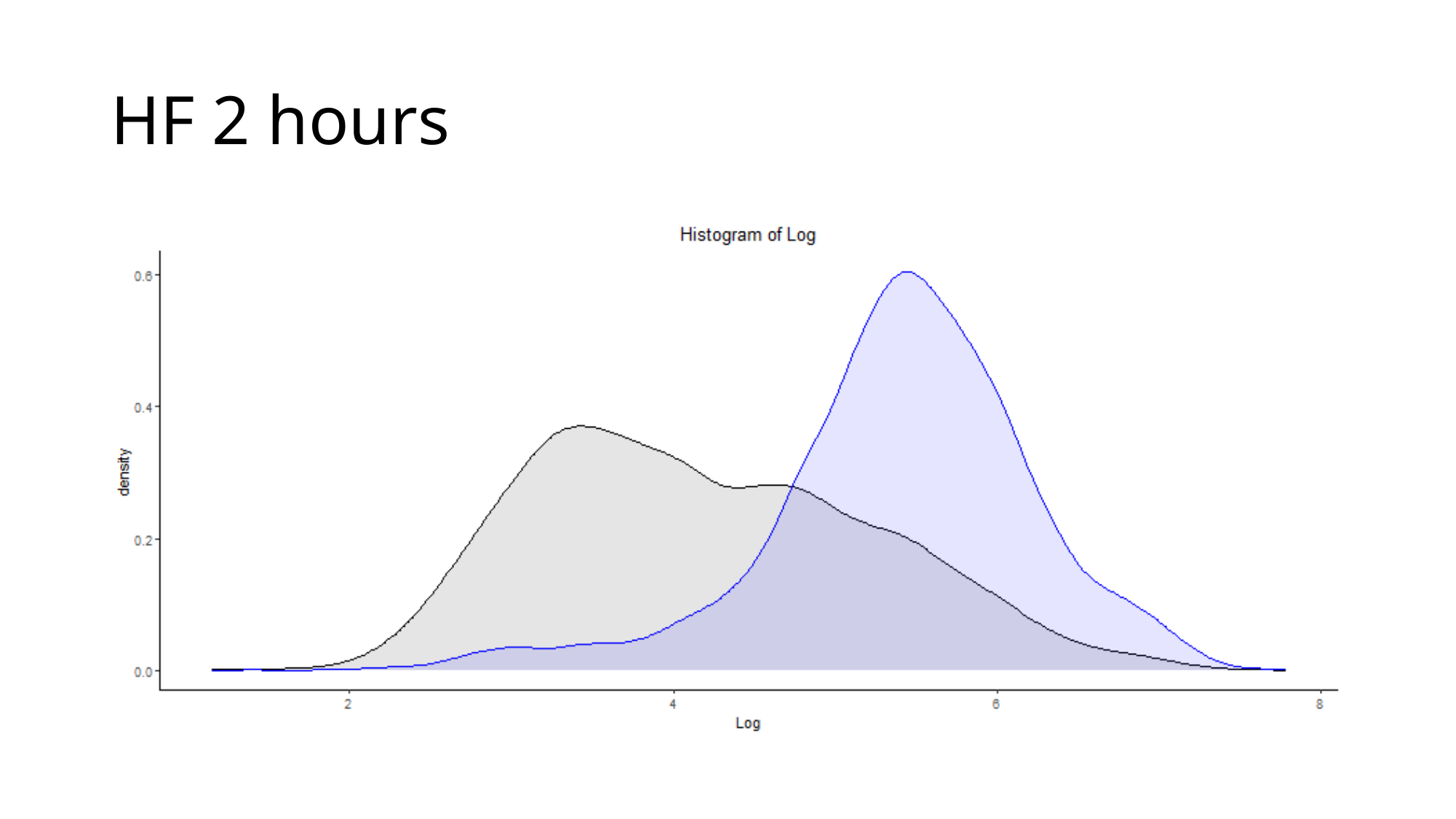

# HF 2 hours

## Slide 32
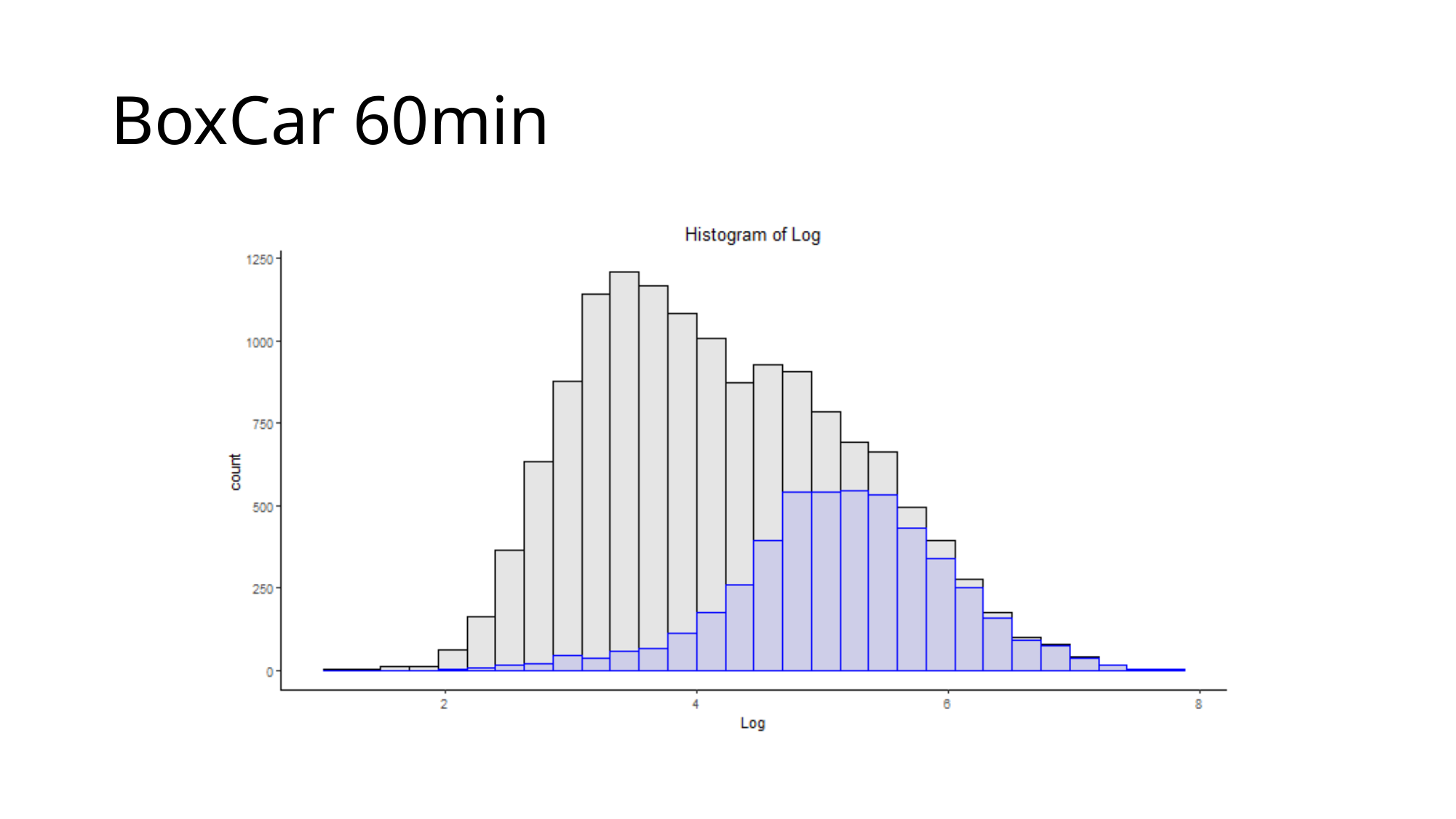

# BoxCar 60min

## Slide 33
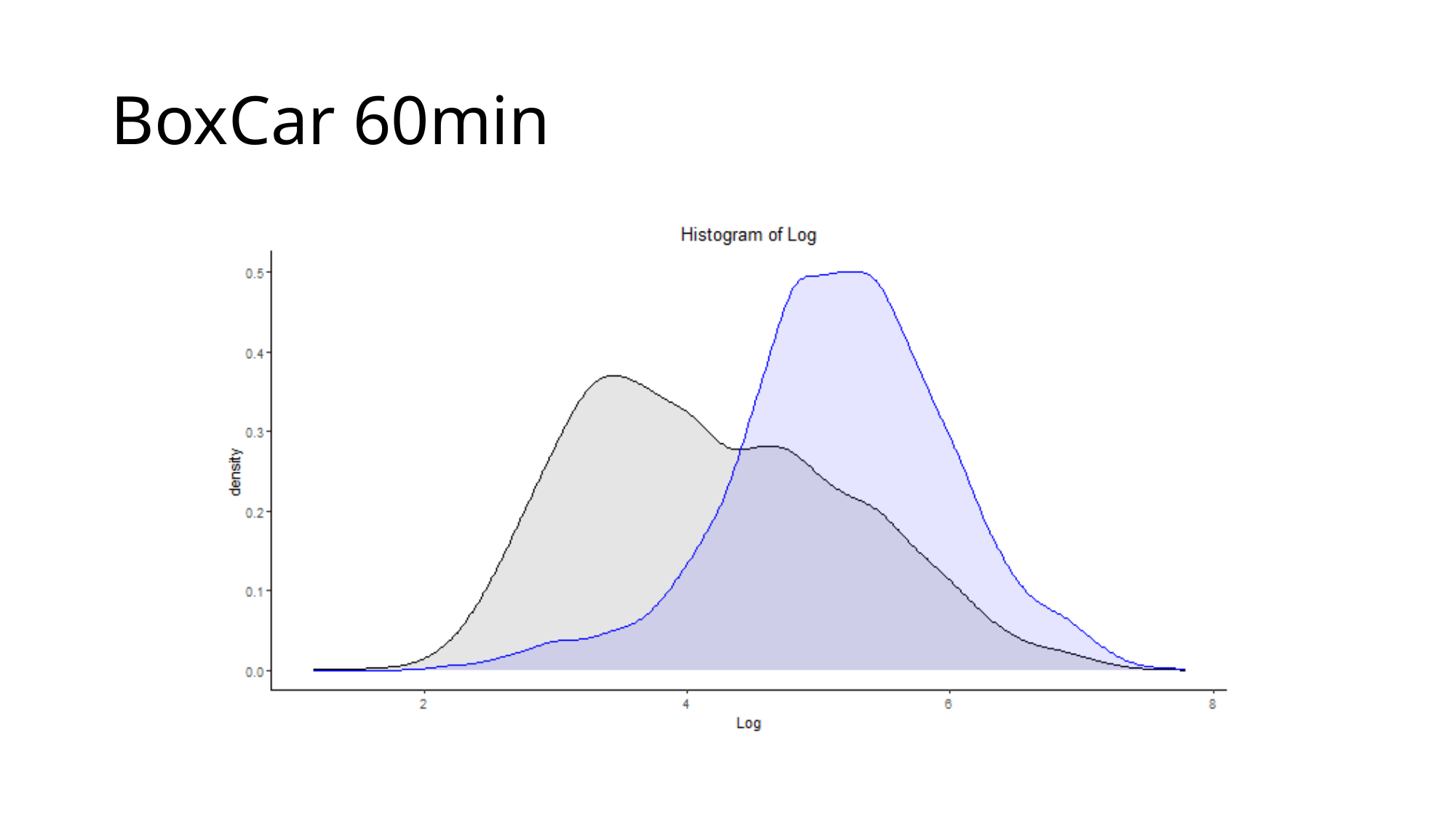

# BoxCar 60min

## Slide 34
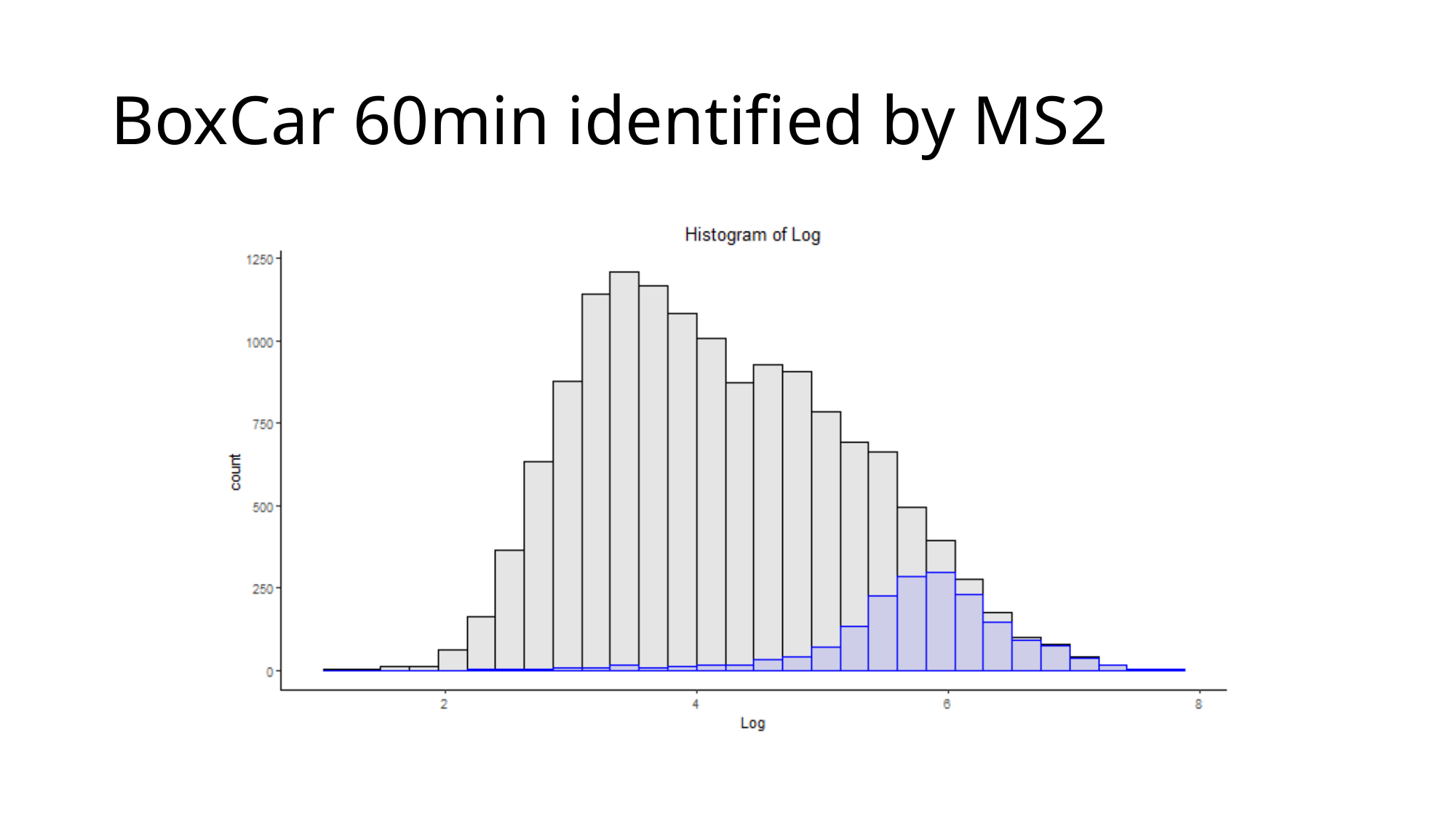

# BoxCar 60min identified by MS2

## Slide 35
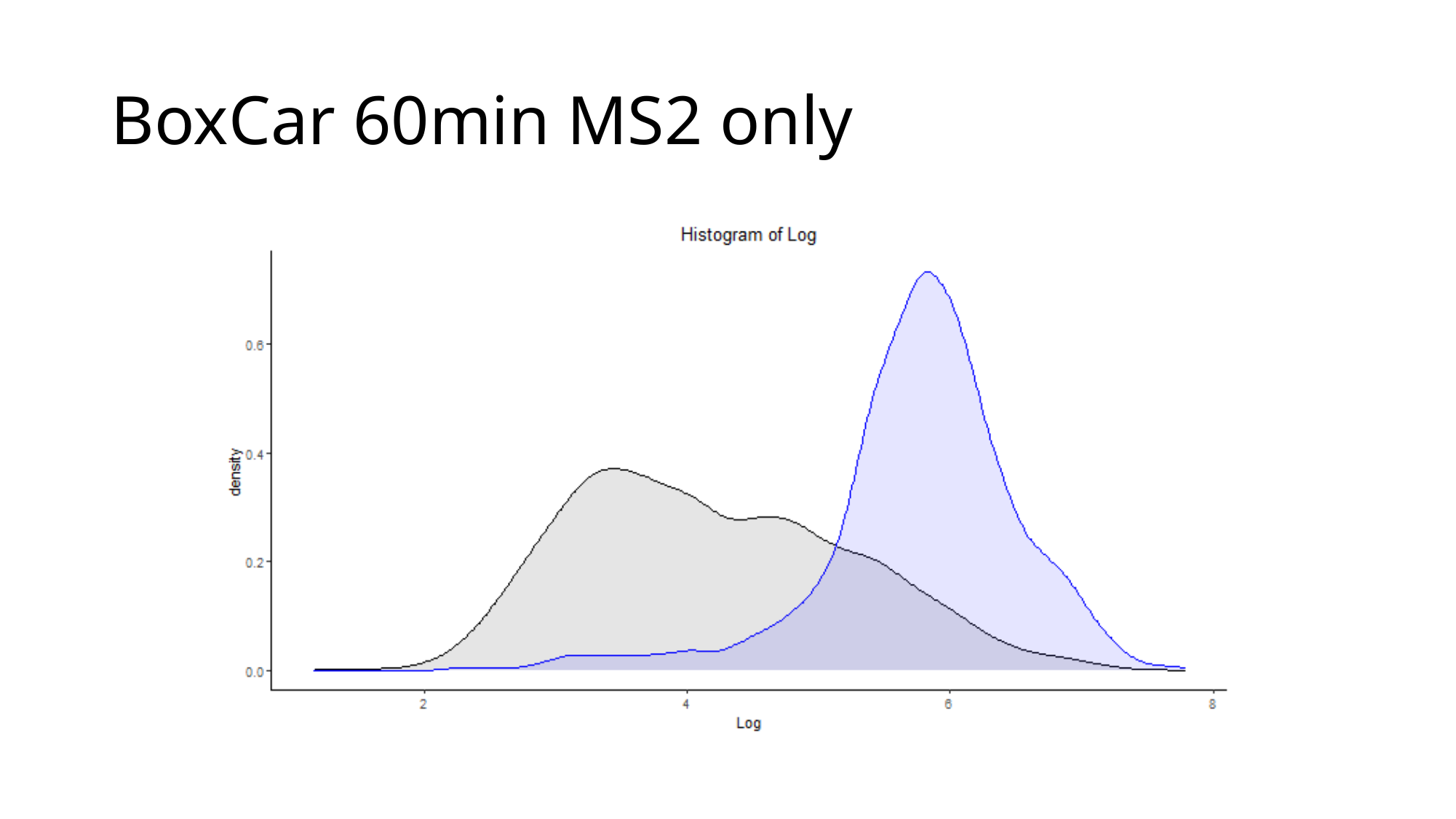

# BoxCar 60min MS2 only

## Slide 36
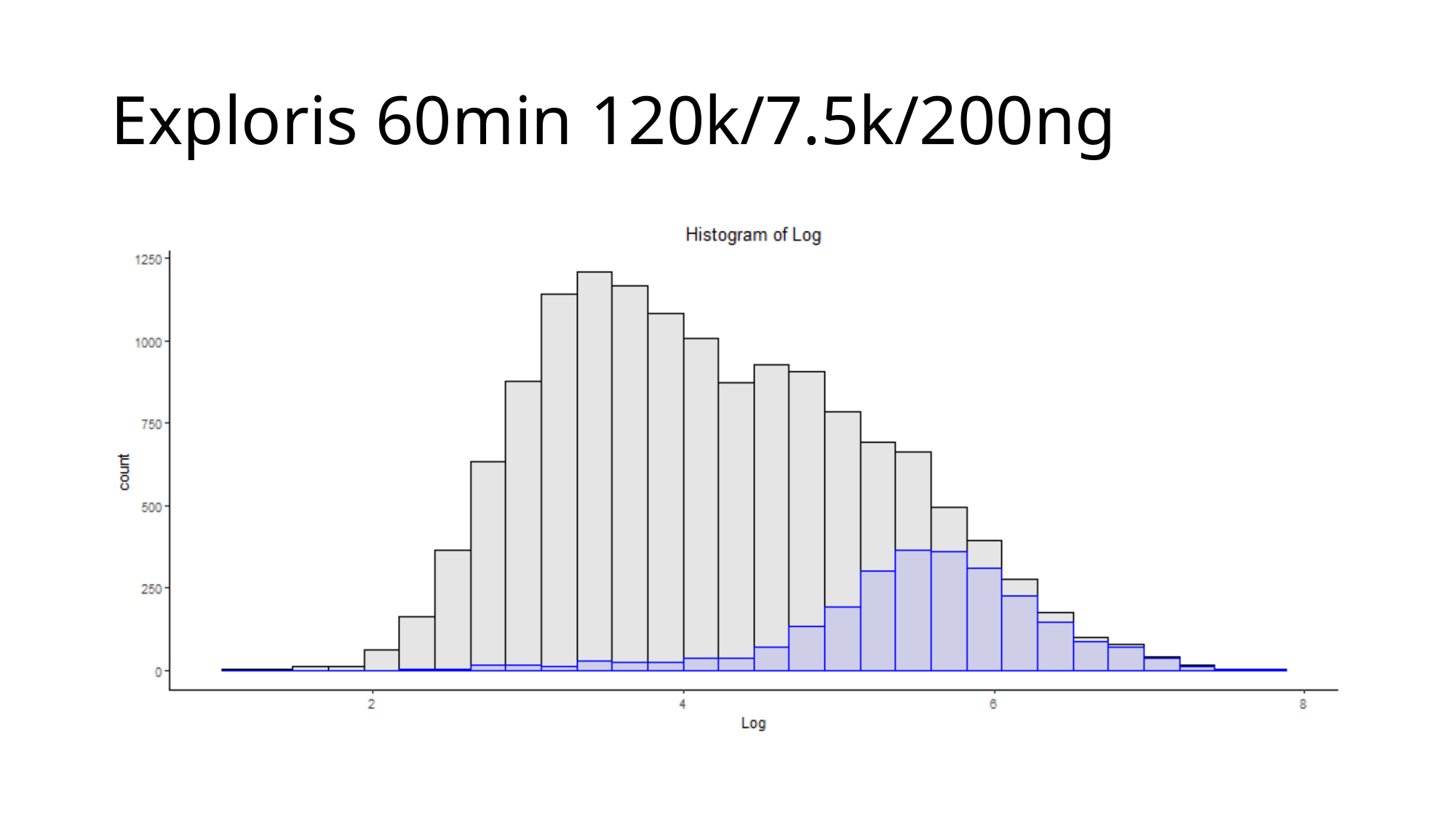

# Exploris 60min 120k/7.5k/200ng

## Slide 37
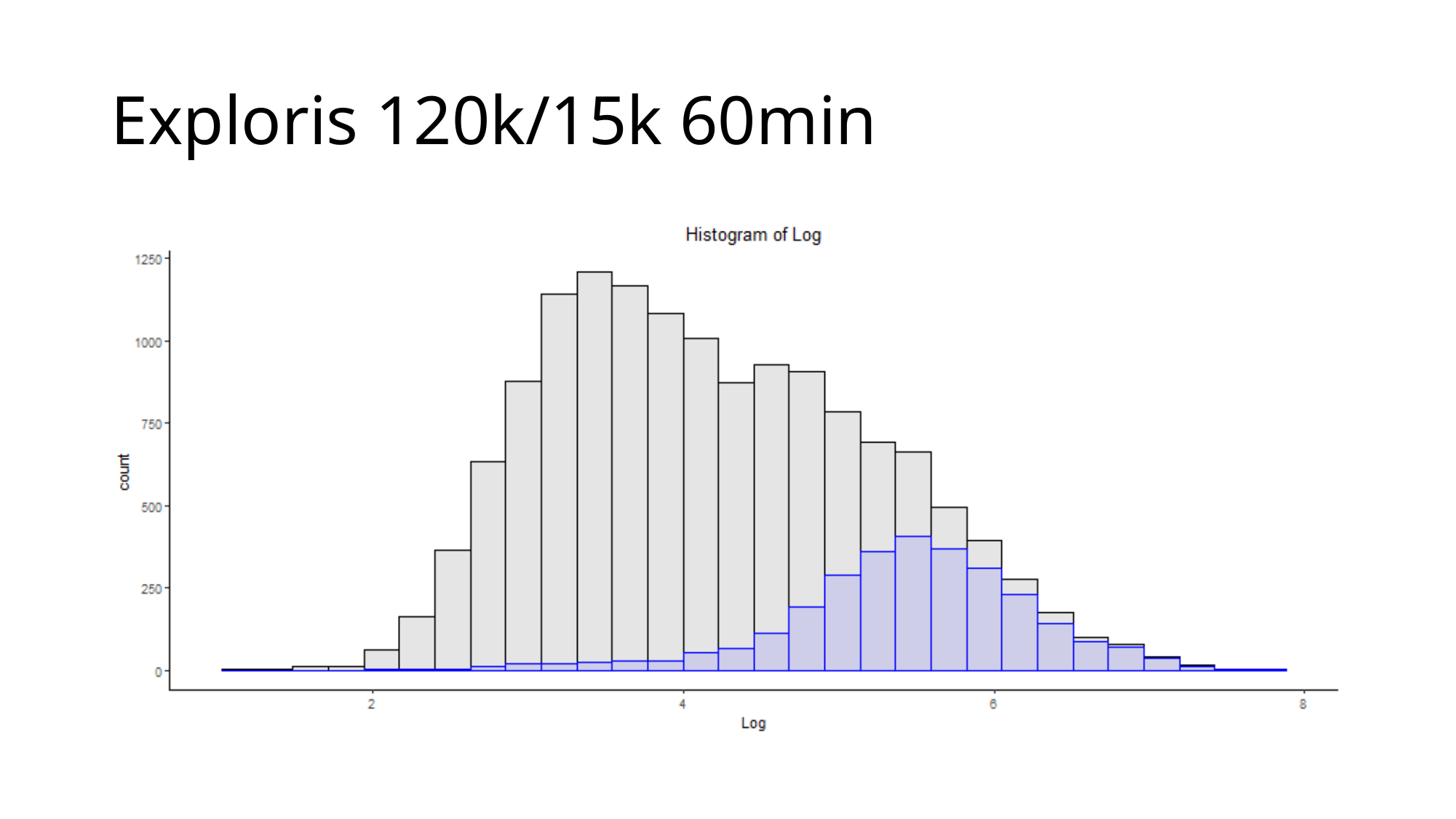

# Exploris 120k/15k 60min

## Slide 38
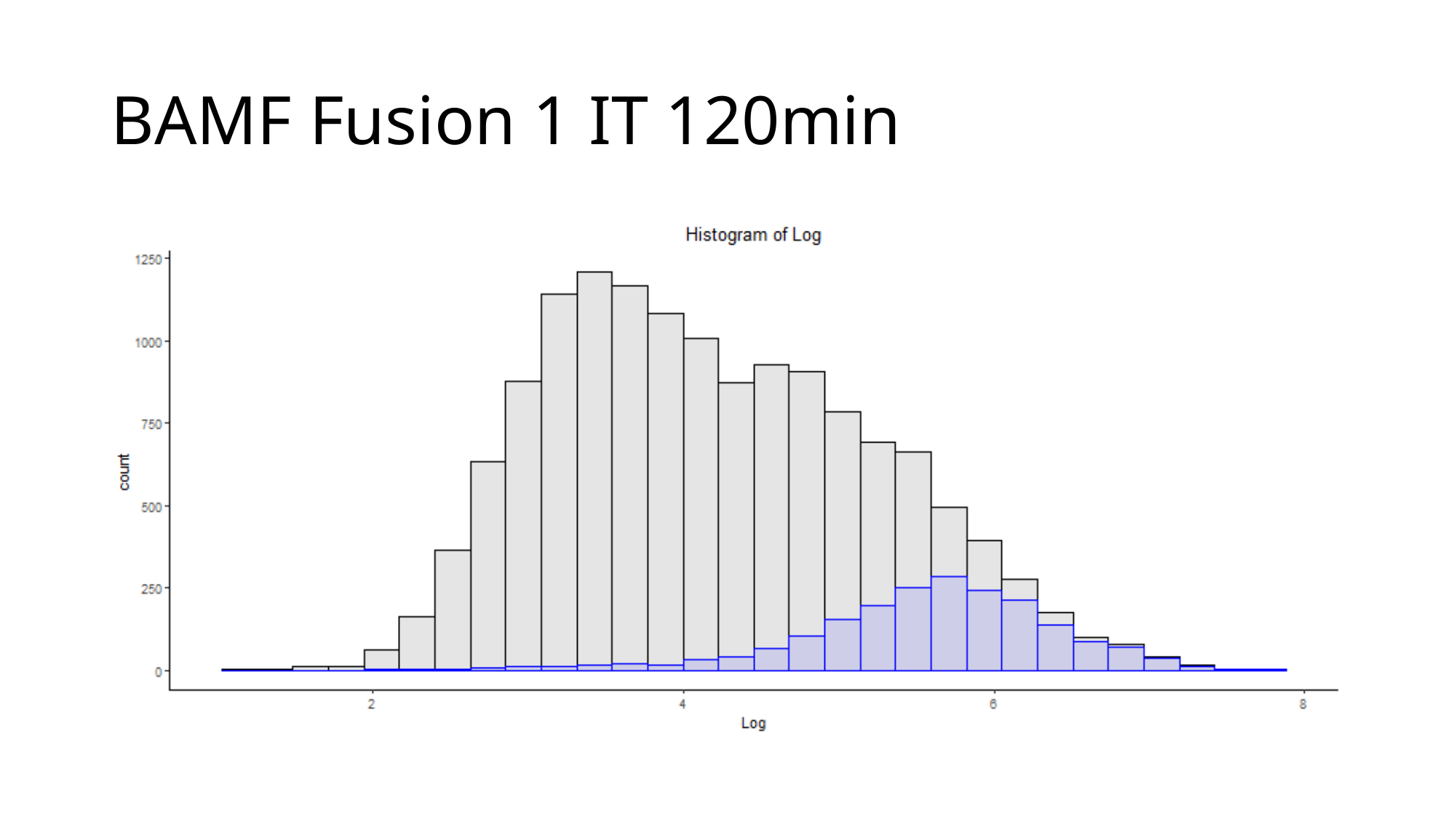

# BAMF Fusion 1 IT 120min

## Slide 39
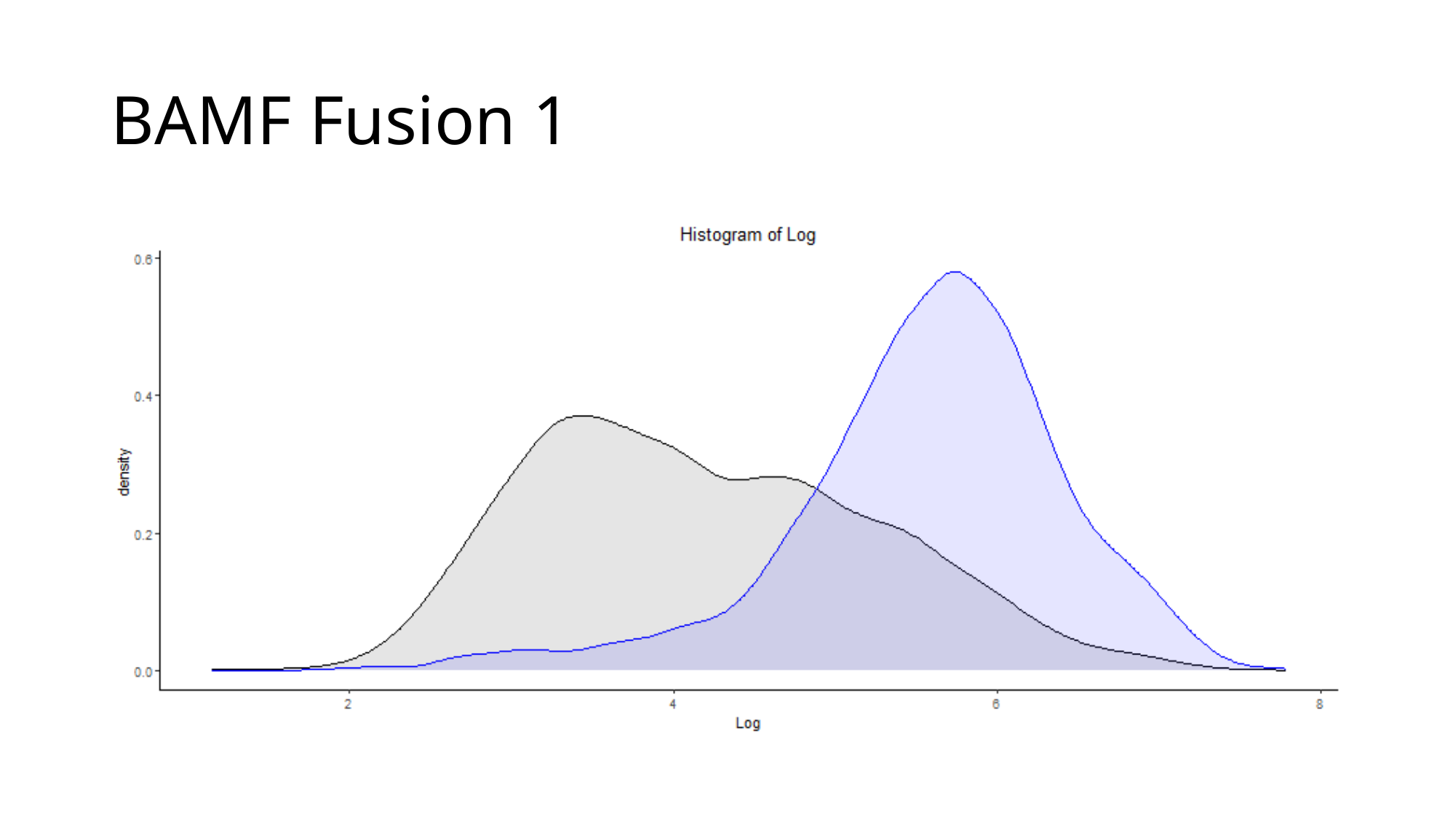

# BAMF Fusion 1

## Slide 40
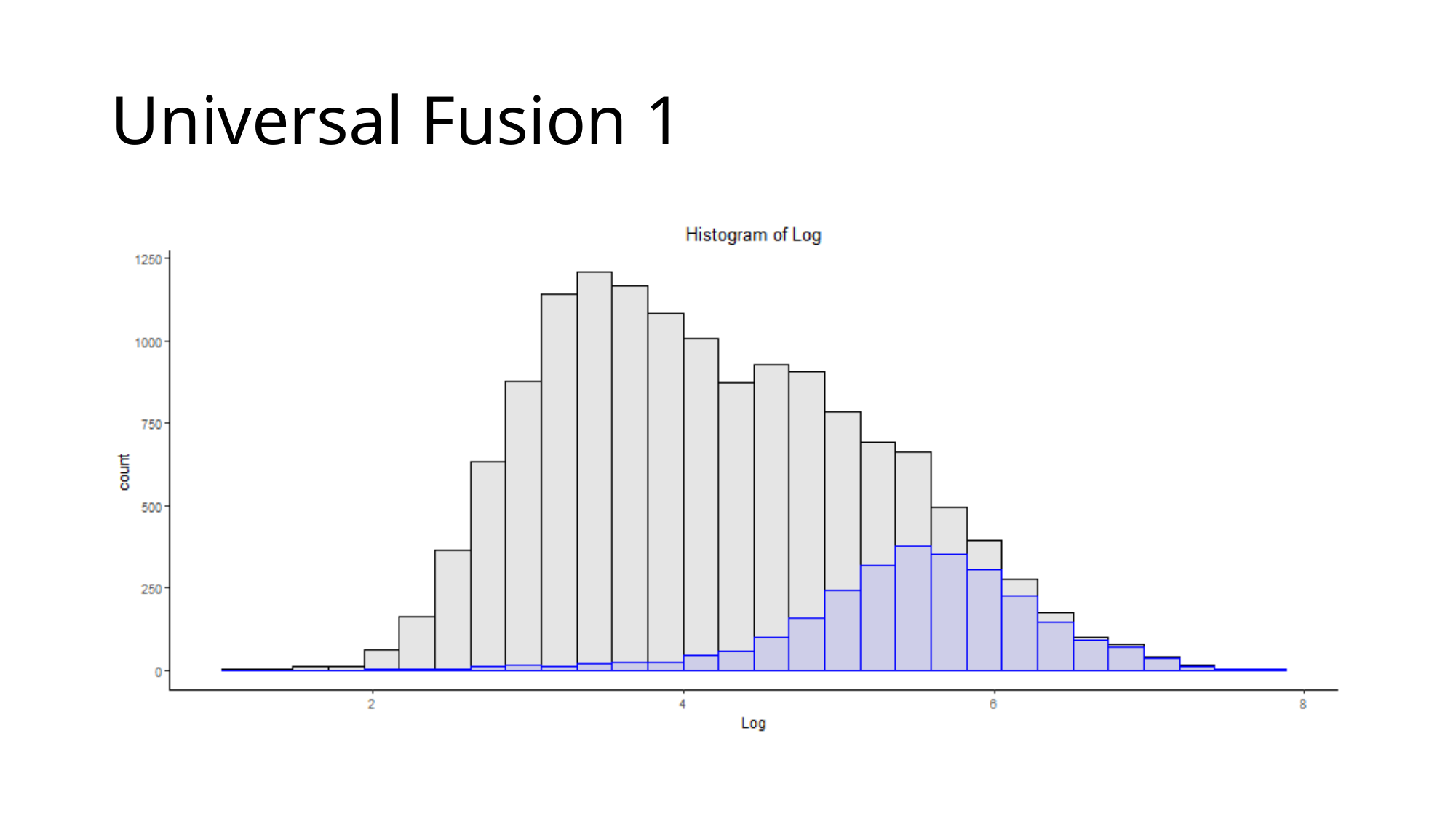

# Universal Fusion 1

## Slide 41
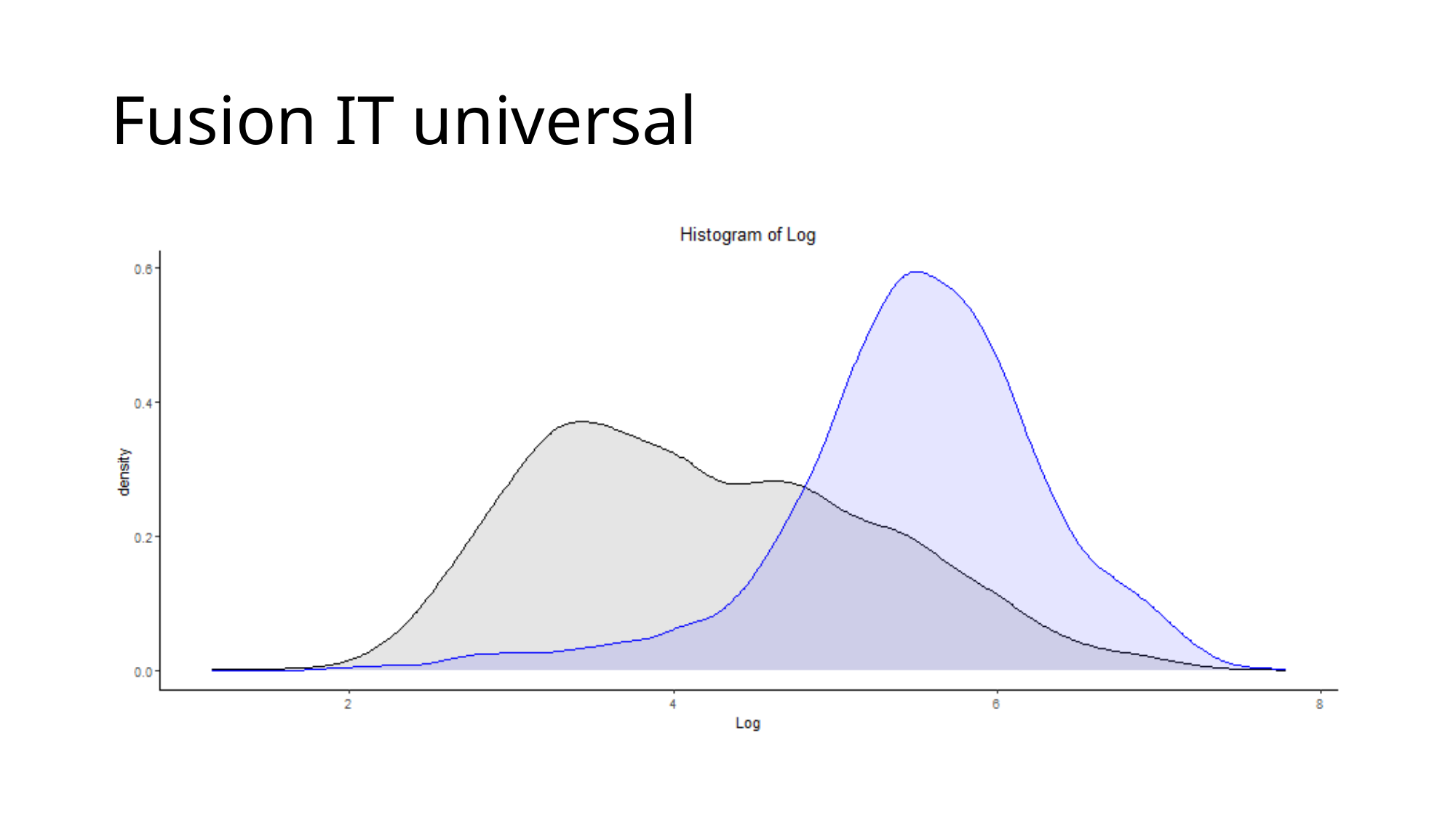

# Fusion IT universal
